# Disclosing Group Members’ Identities Reduces Cooperation in an Artefactual Public Goods Field Experiment

**DOI:** 10.1007/s12110-025-09508-7

**Published:** 2025-11-13

**Authors:** Nils Christian Hoenow

**Affiliations:** 1 RWI-Leibniz Institute for Economic Research, Hohenzollernstr. 1-3, 45128 Essen, Germany; 2 School of Business and Economics, University of Marburg, Am Plan 2, 35032 Marburg, Germany

**Keywords:** Anonymity, Cooperation, Artefactual field experiment, Public good, Social identity, Depersonalization

## Abstract

Social dilemmas in the real world, such as pollution and the extraction of resources, often differ regarding the visibility of involved actors and their behavior. While publicly disclosing individual decisions in social dilemmas is known to increase cooperation, little is known about whether revealing individual identities specifically makes a difference. This study uses an artefactual public good field experiment conducted in rural Namibia with 144 villagers, who are randomly assigned to one of two experimental conditions: one in which group members’ identities are not disclosed and one in which they are. Individual contributions to the public good remain private in both cases, so the only difference lies in whether participants can see who their group members are. In addition, the experiment’s setting in village communities entails pre-existing social ties between participants, which likely amplify potential effects that revealing identities can have on cooperation and allow investigating the role of group composition, such as the share of friends and family members. Results, somewhat unexpectedly, show that contributions to the public good are significantly higher when group members cannot identify one another – a finding that can be explained by several, not necessarily mutually exclusive, mechanisms. Exploratory analyses further reveal that contributions in the identified condition are distinctly lower when group members are socially distant from each other. Although overall variability of contributions does not differ across the two experimental conditions, decisions are more homogenous within groups and more heterogeneous between groups when identities are disclosed.

**Supplementary Information:**

The online version contains supplementary material available at 10.1007/s12110-025-09508-7.

## Introduction

Many of humankind’s major challenges — such as environmental pollution, exploitation of resources, violent conflicts, and certain political decisions, but also numerous everyday social interactions — can be described as social dilemmas, i.e., situations in which individual and collective interests diverge. While the obvious solution to social dilemmas lies in universal cooperation, theory and practice have shown that actors and entities at all levels tend to prioritize their own benefit over the social benefit or overall welfare. Social dilemmas in the real-world often vary regarding the visibility of individuals and their behavior, in many contexts simply due to the large number of people involved.

While publicly revealing individual decisions in social dilemma situations has been found to result in higher cooperation outcomes (e.g., Wichman 1970; Rege and Telle 2004; Savikhin Samek and Sheremeta 2014), they are usually kept private in economic experiments for ethical reasons (Fox and Guyer 1978; Anderies et al. 2011). Experiments are also often conducted with participants who cannot see or identify each other, for example through computer terminals or online (e.g., Fehr and Gächter 2000; Fischbacher et al. 2001; Fischbacher and Gächter 2010). The assumption here is that by keeping individual identities hidden, undesired influences from intragroup dynamics and individual characteristics are minimized or homogenized (Roth 1995; Bohnet and Frey 1999; Andreoni and Petrie 2004). In some lab experiments as well as in many lab-in-the-field experiments, however, settings allow participants to see and identify each other, sometimes they are even given the opportunity to communicate face-to-face (e.g., Dawes et al. 1977; Dubrovsky et al. 1991; Ghate et al. 2013). To date, the distinction between group members in social dilemma situations being identifiable or not has received little consideration and investigation.

In this study, I therefore compare a condition that does not disclose group members’ identities in a public good dilemma with a condition that reveals their identities. In both cases, individual contributions remain private, so that the only difference between the two conditions lies in whether participants get to see who their group members are. The study setting in rural, Namibian village communities was chosen as it entails varying degrees of authentic social closeness between participants, such as being acquainted, being friends or even being related to each other. This is expected to make the effects of revealing identities more salient as participants do not only see and identify unknown strangers, like in lab-experimental settings, but recognize their group members’ identities, which includes the consideration of associated interpersonal relationships.

For several reasons, revealing the identities of group members could be expected to have positive effects on cooperation: First, visual identification of each other may promote the perception of one’s group members as real persons which allows for interpersonal cues and may increase empathy and willingness to behave prosocially (Schelling 1968; Jenni and Loewenstein 1997; Eckel et al. 2001; Majdandžić et al. 2012; Genevsky et al. 2013). If group members can, as in this study’s setting, even be recognized as people with whom one has a personal relationship, such as friends or family members, it will likely further increase the willingness to cooperate (Ben-Ner et al. 2009). This seems intuitive and can, for related individuals, be explained by evolutionary theories, even including relatives of second or higher degree (Darwin 1859; Hamilton 1964; Candelo et al. 2018). Preferences were also found to extend to unrelated but socially close individuals, such as friends (Brewer and Caporael 1990; Gintis et al. 2003; Goette et al. 2006; Henrich and Henrich 2006; Wilson and Wilson 2007; Apicella et al. 2012).

Second, mutual identification can lead to the self-perception as part of a social group, thereby creating a shared group identity, which contributes to favoring the group benefit over the individual one (Tajfel et al. 1971; Billig and Tajfel 1973; Tajfel 1974; Turner et al. 1979; Brewer 1979; Turner et al. 1987; Tajfel and Turner 2004; Balliet et al. 2014). A group identity may be particularly present with socially close group members and may also be promoted through facing a challenging situation, such as a social dilemma, together (Kramer 1989).

Third, the feeling of accountability and responsibility for the group’s outcome may be more salient with real, identified partners, whereas it remains diffused in the non-disclosed condition, similar to a bystander effect (Darley and Latané 1968; Latané and Dabbs 1975; Latané and Nida 1981). A perception of anonymity might also reduce the feeling of guilt for norm-violating behavior and give a sense of protection against social disapproval and punishment (Mann et al. 1982). It has even been argued that anonymity in crowds can lead to deindividuation effects, which include a loss of accountability and self-control associated with impulsive, antisocial and, in the worst case, violent behavior (Le Bon 1896; Festinger et al. 1952; Zimbardo 1969; Fox and Guyer 1977).

While aforementioned theories primarily associate anonymity and respective group dynamics with undesirable behaviors, Reicher et al. (1995) have described a model that predicts potentially positive effects of anonymity. The social identity model on deindividuation effects (SIDE) is based on the self-categorization theory by Turner et al. (1987), which explains how individuals perceive themselves as part of social groups and predicts that individual behavior will be in line with what is seen as the salient norms of the group they belong to (Reicher et al. 1995; Chang 2008). This process is termed depersonalization and differs from what was previously understood as deindividuation in that depersonalization effects can be negative, such as in a group of aggressive rioters or hooligans, or positive, if the prevailing group norms prescribe prosocial or cooperative behavior. According to the SIDE model, anonymity can make the group identity more salient as the group is seen as a homogeneous entity rather than a heterogeneous assembly of individual actors (Lea et al. 2001). Attention is thereby shifted away from the individual and interpersonal towards the group with its common identity, goals, and norms, resulting in a depersonalized perception of the self and others. In order to exhibit any effects, a level of group identity is needed as well as salient, associated group norms to conform with. Finally, the possibility of identification could also lead group members to realize that they do not like or trust each other or increase interpersonal competition, potentially resulting in negative effects of disclosing identities.

Hence, given arguments in favor of both positive and negative effects that the identifiability of group members can have on cooperation, I do not, ex-ante, expect a specific effect direction, but investigate potential differences across the two experimental conditions neutrally using two-tailed tests and regression analyses. With this study, I further test if revealing group members’ identities is associated with more heterogeneous decision behavior or, conversely, if not disclosing identities leads to more homogeneous behavior, which is often seen as methodically desirable for the design of experimental studies (Roth 1995; Bohnet and Frey 1999; Andreoni and Petrie 2004). The setting in village communities also allows exploring the effects of social closeness on cooperation, especially so within the identification condition and thereby adds to the research on cooperative behavior with socially close and even related individuals (Handley and Mathew 2020). Especially for the latter, a strong positive association is often assumed, but has sparsely been shown empirically (cf. e.g., Peters et al. 2004; Ben-Ner et al. 2009).

In the past, a few studies similar to mine have been conducted; yet they focus on student samples, and their findings are not concordant. Wichman (1970) played a repeated prisoners’ dilemma that included an anonymous and a see-each-other condition with 22 female students per experimental group. Cooperation was initially higher in the anonymous group, but in later rounds the see-each-other groups surpassed the anonymous ones. Since there were only two players in their prisoners’ dilemma, also individual decisions were (indirectly) revealed. Bohnet and Frey (1999)conducted a 4-player prisoners’ dilemma with 240 undergraduate students, who did not know each other beforehand, and found more cooperation when group members could be identified. Brosig and Weimann (2003), on the other hand, found no differences between an anonymous and a silent-identification condition in their study which consisted of 20 undergraduate students per experimental condition. Butz and Harbring (2021) more recently conducted a public good lab experiment in which group members of one condition were told that they would get to meet their group members after making their decisions. No general effect of this subsequent disclosure on contributions was observed in comparison to an anonymous condition. In contrast to Butz and Harbring (2021)as well as Bohnet and Frey (1999) and Brosig and Weimann (2003), who emphasize that they deliberately aimed to avoid previous acquaintanceships among their participants, my study explicitly considers pre-existing social ties between village community members. This setting is expected to make effects of disclosing identities more salient. Indeed, Sampaio et al. (2023) observed that information on group members’ past cooperative behavior, i.e., their reputation, can interact with cues on their identities, such as names and photographs.

## Research Setting and Method

The experiments were conducted during austral winter 2017 in ten randomly selected rural villages in the Kapako district (Kavango West) and in the Ndiyona district (Kavango East). They were embedded in a larger study on cooperation, deforestation and development in rural Kavango, which, in turn, was a part of the SASSCAL research project on climate change and adaptation. For this study, I specifically make use of pre-existing social ties, both kinship and friendship, among participants from rural village communities.

### Study Setting

The Kavango regions are home to over 350,000 people living across nearly 50,000 square kilometers (Pröpper et al. 2015; Namibia Statistics Agency 2023a, Namibia Statistics Agency 2023b). Despite the presence of schools and some healthcare facilities, the area remains overall underdeveloped, with widespread poverty and few employment opportunities (Pröpper et al. 2015). The population is growing rapidly, with increasing urbanization, particularly among younger residents moving toward urban centers such as Rundu. The region is experiencing a social and economic transition, as aspirations for a more modern, market-oriented lifestyle make cash incomes and access to consumer goods increasingly important, reducing the perceived reliance on traditional resources and subsistence farming. Social stratification is prevalent, with some households benefiting from successful smallholder farming or paid employment, while others lack both financial resources and productive assets.

Among villagers, smallholder agriculture remains the predominant livelihood and is mostly subsistence-based crop farming, largely reliant on manual labor. Cattle farming plays a secondary but notable role (Namibian Ministry of Lands and Resettlements 2015). Farming and resource use can be described as common-pool dilemmas: forests are cut for crop land through slash-and-burn practices, but also provide timber, firewood, game, herbs, and fruits – while intact. Low soil fertility limits long-term cultivation unless fertilizers are used, which is rare, and competition for land and resources is increasing, particularly so near the densely populated riverside. Collective management of natural resources is facilitated by traditional authorities, who are generally well accepted within villages and exercise some control over land allocation and are called upon to resolve conflicts, while higher-level authorities coordinate across larger areas (Hinz 2003; Pröpper et al. 2015). There are village meetings as well as social and religious gatherings held regularly, and some households work together in agricultural tasks. Kinship relations are present across many households as well as across villages and are seen as important. While villagers tend to live in the same place for many years or even a lifetime, migration from and to village is also common, driven by marital ties and the search for fertile land further inland, away from the densely populated riverside areas. The population is linguistically diverse, with several dialects spoken and a small presence of San people, some of whom have been integrated as villagers. Religion is predominantly Christian, though traditional spiritual practices persist alongside formal faiths.

### Data Collection and Study Sample

**Fig. 1:**
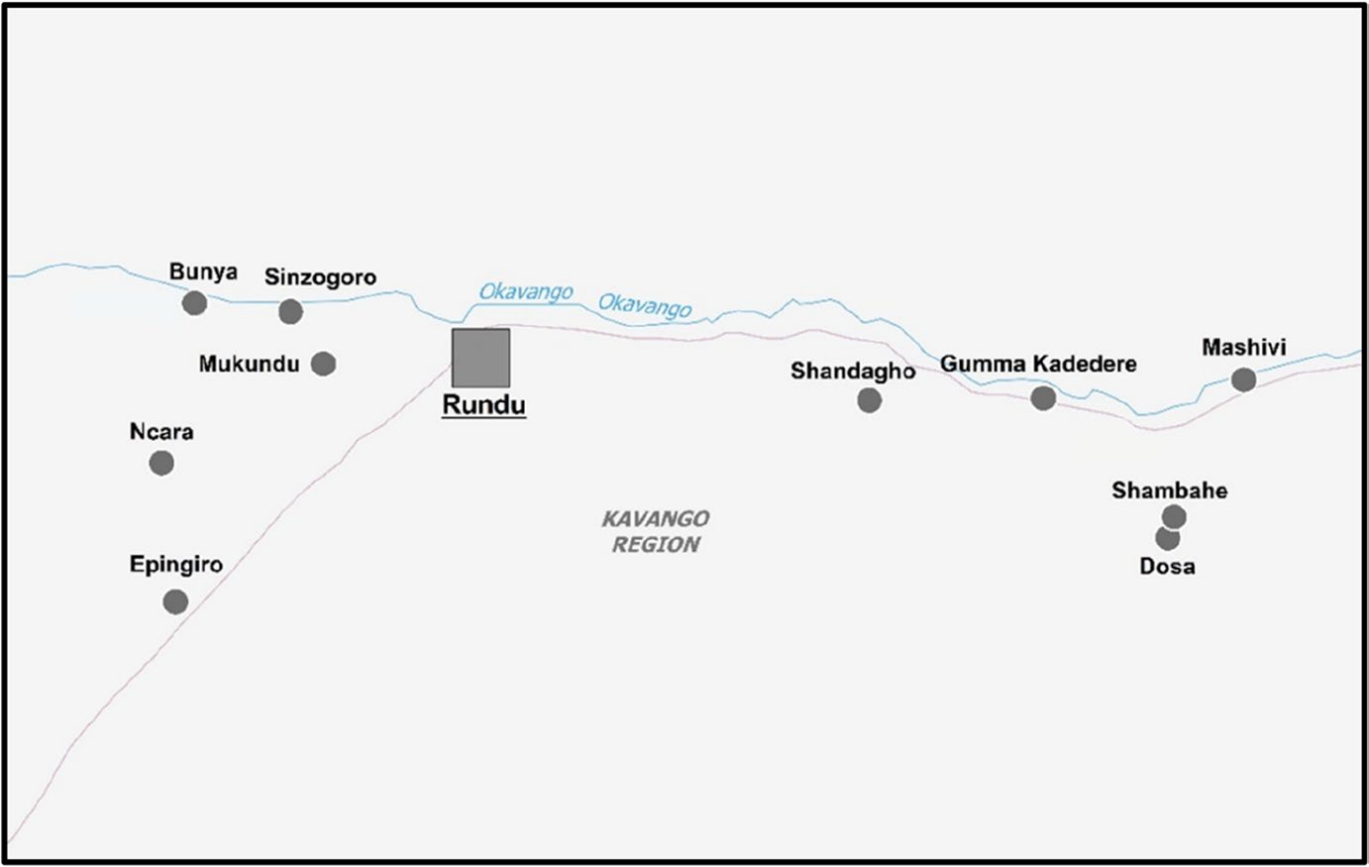
Selected villages in the Kavango regions

The study was conducted from April to June 2017 in ten villages of the Kavango regions in Namibia, the names and the positions of which are shown in Fig. 1. For the selection, villages that had formerly been visited for similar research projects were left out. Other preconditions were that the selected villages were within a day’s drive of the nearest paved road and had more than 80 inhabitants, considered a sufficiently large number for random sampling. The visited villages varied in size and population with a mean of 823 and median of 300 inhabitants. The total sample size of the data used in this study is 144 participants, 72 in each of the two experimental conditions.

In preparation of the experimental workshops, each village’s headperson was visited several days in advance to arrange an appointment for a village meeting so that all villagers could be informed and invited in time. It was made clear beforehand that some monetary compensation would be offered for participating but also that only a certain number of participants would be able to take part in the workshops. At the beginning of each village meeting, 24 participants were randomly drawn by lot amongst those who expressed willingness to participate. This selection procedure was considered fair by almost everybody. The same lots also determined the allocation to one of two experimental conditions per village. These experimental conditions consisted of 12 players each, who were then spatially separated. The experimenters explained to them the procedure of the workshop as well as the instructions of the public good game according to the respective experimental condition.^1^

Protocols and instructions had previously been translated by the local assistants from English into the respective local languages and then translated back into English by another assistant to ensure that all translated instructions were on point. Also, all wordings and phrases used in the instructions were discussed intensively with local assistants in preparation of the experiments as to make all instructions clear and easily understandable. Each experimental condition was supervised by one experimenter and one local research assistant for interpretation. Local assistants were recruited in the town of Rundu and remained the same team over all visited villages, while the allocation of assistants and experimenters to experimental conditions was randomized for each village.

The collected sample consists of 53% women, with a mean age of 36 years and an average education of 6.6 years of schooling. The average household size is 4.6 adults and 4.0 children. Agriculture is the primary profession for 87% of participants, with households cultivating an average of 2.99 hectares, producing 8.3 bags (50 kg each) of crops per year, and earning 726.92 NAD annually from crop sales. Livestock ownership was common, with households keeping an average of 10 cattle, and total annual household income across all sources averaged 10,797.94 NAD (median: 3,000 NAD). Regarding migration, 53% of participants were born in their current village, 11% moved there more than 20 years ago, 21% moved 11–20 years ago, 9% moved 6–10 years ago, and 6% moved within the past five years. The religion of the sample is overwhelmingly Christian, with 95.8% reporting some form of Christian affiliation. Local dialects reflect regional differences, with 47.6% speaking Rukwangali (mostly in Kavango West) and 41.3% of participants speaking Gciriku (mostly in Kavango East) as their mother tongue, smaller percentages speaking other dialects, and 2.1% are native speakers of a (Khoi-)San language.

### The Public Good Experiment

**Fig. 2:**
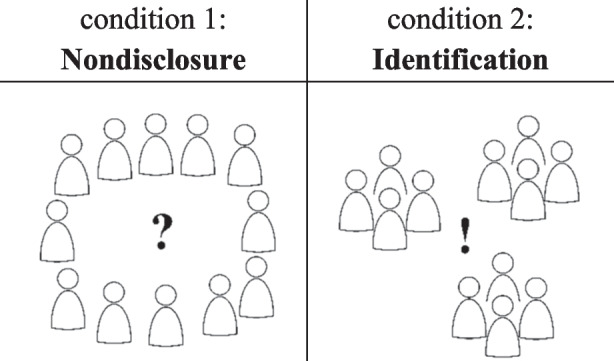
Illustration of experimental conditions

To elicit cooperative behavior, I conducted a single-round, unframed public good experiment with four players per group. The game was incentivized with real money and players could make contributions to the public good between 0 and 10 experimental coins. Collected contributions then got doubled and distributed equally amongst all group members. Experimental coins were later converted to 5 Namibian Dollars (N$) each. Details about the public good game can be found together with the experimental protocols and the game instructions in the Supplementary Materials (A.1, B). I randomly allocated participants to either an experimental condition that did not disclose the identities of group members or to another one that revealed them. The individual contributions to the public good remained confidential in both cases. There were always three groups of four players per experimental condition and village. Hence, in the nondisclosure condition, players could see and identify 11 potential group members in their part of the workshop but were informed that they would not become aware of their three partners’ identities, not even after the experiment.^2^ In the identification condition, participants were seated together with their group members and told that those allocations were the groups playing the public good game together. Figure 2 illustrates the concept. Communication of any form was prohibited in both conditions.

To check whether participants understood the public good game players were asked two control questions before making their decisions (Supplementary Materials B.9 for exact wording). Out of the total 144 participants, 114 answered both of those questions correctly, which is denoted in the following as “cqc” (control question correct) when focusing on this subsample. Participants were also asked in private about their expectations (often termed “beliefs”) of their three group members’ average contributions (Supplementary Materials B.9). This elicitation was also incentivized: Participants who correctly guessed their group members’ average contributions (within a range of ± 1 experimental coin) received a bonus payment of 20 N$. Decisions were then made sequentially in a secluded place, such as behind a building, using differently colored envelopes that represented the private and the group account. To visualize the process, the envelopes with contributions to the public good had to be put in a common basked, whereas the envelopes that contained the remaining amount were kept by the participants. This procedure ensured confidentiality of decisions, not only in front of one’s group members but also towards the experimenters as players, envelopes and payments were only handled through randomized ID-numbers. After all players had made their decision, socioeconomic characteristics as well as (stated) information on the group composition, specifically on the number of friends and family members in one’s group were collected in individual interviews (Supplementary Materials C). As family I counted relatives and anyone living in the same household. Since participants only sometimes stated that some of their group members were strangers, I grouped “acquaintances” and “strangers” together as the counterfactual category to “family” and “friends”. While for the identification condition the variables for friends and family members were elicited on group level, groups were not revealed in the nondisclosure condition, and I therefore asked for the number of friends and family members in the whole experimental condition group of 12 players. To make the measurements comparable, numbers shown for social ties in the nondisclosure condition are reported numbers divided by eleven (other workshop participants) and multiplied by three (number of players per group, excluding oneself). The survey questions asked in the post-experimental interviews are shown in the Supplementary Materials (C).^3^ The average payoff was at 75 N$, which equaled about 6 US$ and was worth more than a day’s wage for farmwork in the region. Payments ranged between a minimum of 25N$ and a maximum of 145 N$ (~ 2US$ and 11 US$), including the bonus payment for correctly guessing one’s group members’ average contribution. Table 1 summarizes the variables used in the subsequent analysis. A comparison of socioeconomic characteristics across the two experimental conditions and tests for equality of the subsamples can be found in the Supplementary Materials (A.2).

**Table 1:** Summary statistics and variable description

variable	obs	mean	sd	min	max	variable info
contribution	144	3.88	3.70	0	10	contribution to public good in experiment coins
contribution (cqc)	114	3.88	3.71	0	10	contribution to public good in experiment coins
expectation	144	5.99	2.83	0	10	expectations of others’ average contributions
expectation (cqc)	114	5.96	2.84	0	10	expectations of others’ average contributions
control q. wrong	144	0.21	-	0	1	wrong answer in one or both control questions
friends (workshop)	72	0.65	0.67	0	3	number of friends in workshop
family (workshop)	72	0.77	0.76	0	3	number of relatives in workshop
friends (group)	71	0.77	1.07	0	3	number of friends in group
family (group)	71	1.43	1.25	0	3	number of relatives in group
age	143	35.85	14.14	17	84	age of participant in years
female	143	0.53	-	0	1	gender (1 for female)
schooling years	143	6.59	3.55	0	17	years of schooling
social ladder	143	2.48	1.95	1	10	self-assessed status in village
hectares (log)	143	1.21	0.57	0	3.04	hectares of fields currently cultivated (log)
cattle (log)	143	1.42	1.48	0	4.74	number of cattle owned (log)
migrant	143	0.06	-	0	1	moved to village less than 5 years ago

cqc = only subsample of those who correctly answered both control questions for understanding (*n* = 114). Socioeconomic details of one participant are missing as he left the workshop after the game and could not participate in the post-experimental survey interview

### Method of Analysis

**Fig. 3:**
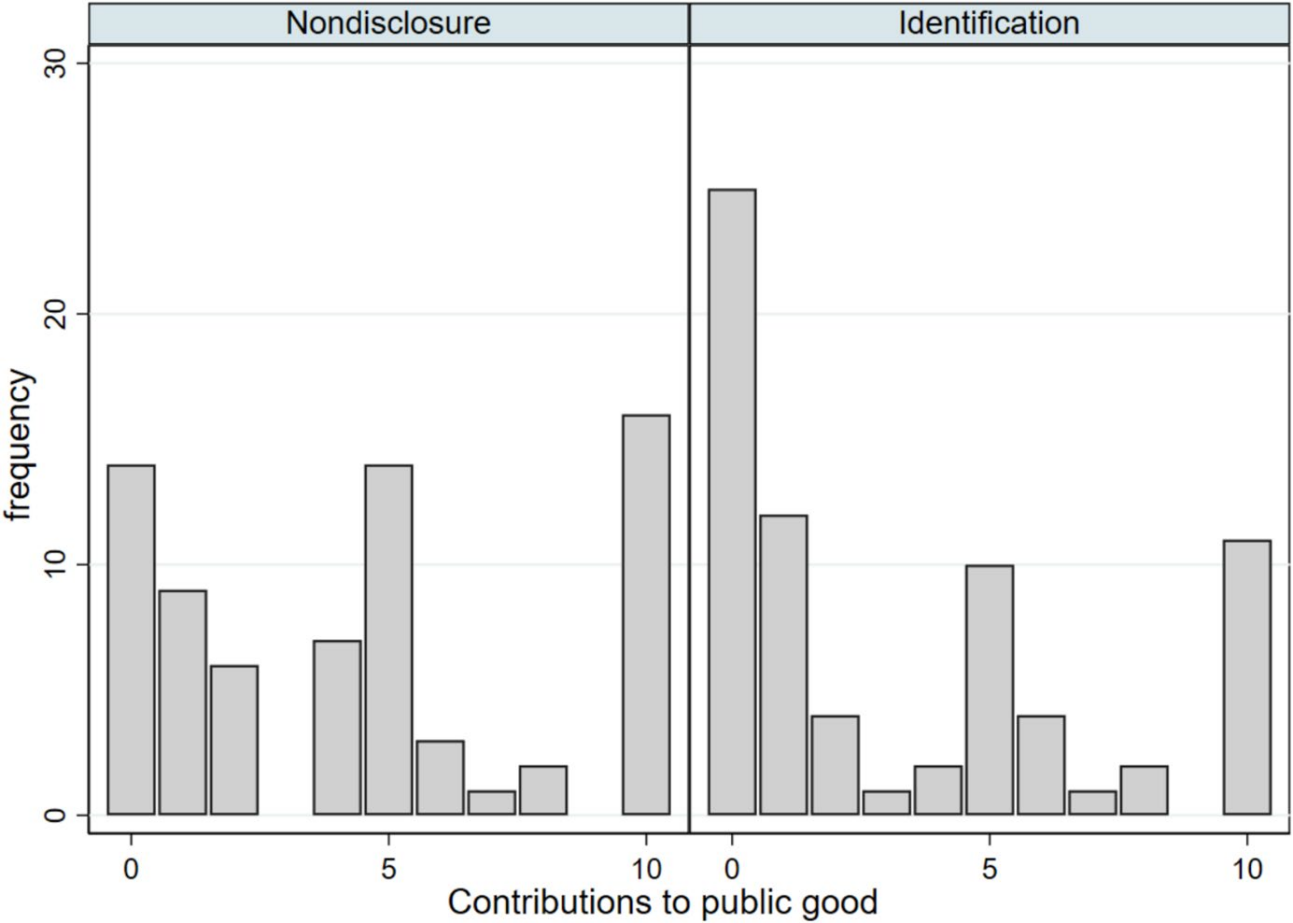
Distribution of contributions

For intuitive understanding, results are first presented graphically, by showing simple averages across experimental conditions and the corresponding differences. In a second step, analyses are complemented by multilevel ordered logit regression models. Given the trimodal distribution of contributions observed across experimental conditions (Fig. 3), the dependent variable is recoded into three categories: 0–1 coin as “low,” 2–8 coins as “some”, and 9–10 coins as “much”.

Random intercepts are included at both the village and group (of 4 players) levels to account for potential clustering, which could otherwise bias standard errors and inference. This approach reflects the assumption that participants’ behavior within villages may be more similar than between villages, with the same logic applying to groups playing together, specifically in the identification condition.^4^ By including varying intercepts, the model simultaneously accounts for correlated residuals within the same entities. While the main specification allows for varying intercepts, random slopes for the effect of condition across villages are considered as robustness checks (Supplementary Materials A.4), as the impact of nondisclosure versus identification may differ between villages.

Results are reported as odds ratios, which indicate the change in the odds of moving into a higher category of contributions, holding other variables constant. As model variations, standard socioeconomic controls are included to assess model robustness, and additional analyses focus on the subsample of participants who correctly answered both comprehension control questions.

## Results

### Contributions to the Public Good

**Fig. 4:**
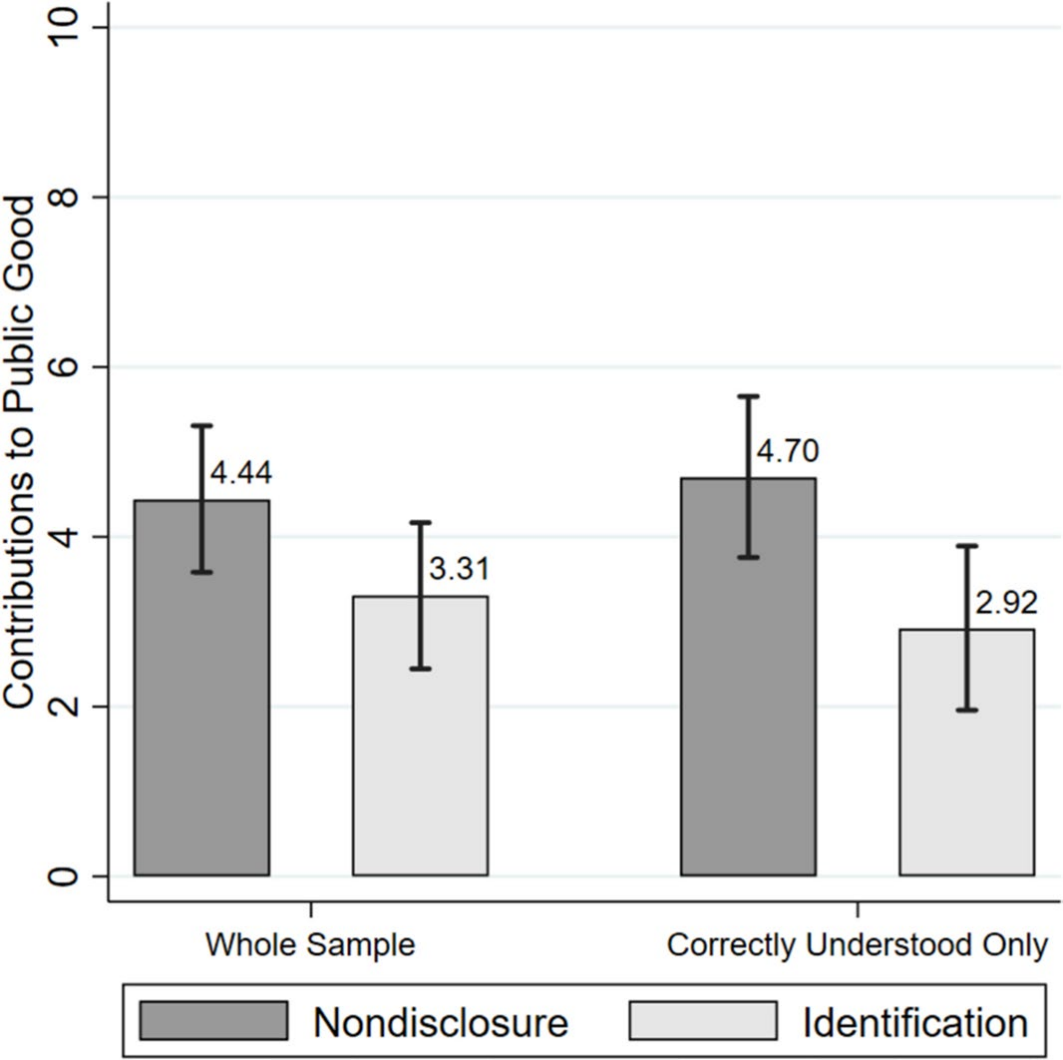
Average contributions by experimental condition. Note: Bars indicate mean contributions, and whiskers show 95% confidence intervals. Whole sample is based on all 144 participants, whereas the subsample of those who correctly answered both control questions for understanding consists of 114 participants

As shown in Fig. 4, average contributions were higher in the nondisclosure condition (4.44 coins) than in the condition with revealed identities (3.31 coins), resulting in an average difference of 1.14 coins (Table 2). When focusing on the subsample of those participants who have correctly answered both control questions for understanding, the difference becomes larger (1.78 coins) and more significant. 5

**Table 2:** Contributions and expectations by experimental condition

**experimental**		**1**	**2**	**Δ**	**t-test**^a^
**condition:**		**nondisclosure**	**identification**	difference	for equality
	n	mean (std. dev.)	mean (std. dev.)	means (std. err.)	p-value
contribution	144	4.44 (3.67)	3.31 (3.66)	−1.14 (0.61)	0.065*
contribution (cqc)	114	4.70 (3.70)	2.92 (3.51)	−1.78 (0.68)	0.009***
expectation	144	6.01 (2.89)	5.96 (2.80)	−0.06 (0.47)	0.907
expectation (cqc)	114	6.10 (2.89)	5.79 (2.80)	−0.31 (0.53)	0.568

cqc = only subsample of those who correctly answered both control questions for understanding. An extended table including more variables can be found in the Supplementary Materials (A.2). Two-tailed t-tests, significance levels: * p < 0.10, ** p < 0.05, *** p < 0.01

As Fig. 3 has shown, distributions within both experimental conditions are distributed roughly trimodally. Multilevel ordered logistic regressions were therefore calculated with random intercepts on village and playing group level (Table 3). Odds ratios are shown for effects sizes.

**Table 3:** Effects of revealing group members’ identities

DV: contributions	**model 1**	**model 2**	**model 3**	**model 4**
	odds-ratios *(p)*	odds-ratios *(p)*	odds-ratios *(p)*	odds-ratios *(p)*
T: Identification	0.301*	0.269**	0.203**	0.197**
	*(0.05)*	*(0.03)*	*(0.02)*	*(0.01)*
*include covariates*	*no*	*yes*	*no*	*yes*
*exclude misunderstood*	*no*	*no*	*yes*	*yes*
N	144	143	114	113

Multilevel ordered logit regressions with random intercepts for villages and playing groups. Odds ratios shown for effect sizes. The dependent variable, contributions, is trichotomized into three categories: 0–1 coin (“low”), 2–8 coins (“some”), and 9–10 coins (“much”). Models 1 and 2 show results for the whole sample, whereas models 3 and 4 focus on participants who have correctly answered both control questions for understanding. P-values are shown in parentheses. Significance levels: * *p* < 0.10, ** *p* < 0.05, *** *p* < 0.01

Overall, there is a consistent negative effect of disclosing identities on contributions, corresponding to a 70–80% lower likelihood of being in a higher contribution category in the identification condition. Conversely, participants in the nondisclosure condition have roughly three- to fivefold higher odds of contributing at a higher level. The magnitude and statistical significance of this effect increase when socioeconomic covariates are included and when restricting the sample to participants who correctly answered both control questions, which is consistent with the results obtained from simple mean comparisons.^6^ The full regression tables with logit coefficients and effects of all covariates (model 2 and 4) can be found in the Supplementary Materials (A.3). None of the added variables for socioeconomic characteristics have a significant effect on contribution decisions. Allowing for random slopes across villages for the effect of the identification condition yields overall similar estimates, albeit with slightly reduced statistical significance (Supplementary Materials A.4).

### Variability of Contributions

While the average contributions differ, the overall variability, measured in standard deviations of contributions, is almost the same in both experimental conditions (Table 2). Looking into variability in more detail, I find that the mean of standard deviations within each group is slightly larger in the nondisclosure condition (3.08 vs. 2.44), but the standard deviation of group means, i.e., the variability across groups, is a little smaller in the nondisclosure condition as compared to the identification condition (2.32 vs. 2.75, for a table, see Supplementary Materials A.6.2). This seems intuitive considering that social ties between participants may affect decisions to contribute on group level. As an additional measure of variability, F_ST_ statistics are calculated for the variation of contributions within and between playing groups (of 4 players) for each experimental condition (e.g., Bell et al. 2009; Smith et al. 2018; Beheim and Bell 2024). These also show a larger share of between group variation (54.11%) in the identification condition, reflecting lower within-group variation, as compared to the nondisclosure condition (38.19%), which confirms above results.

### Expectations

The expectations of one’s group members’ contributions can, with caution, be interpreted as a measurement of (descriptive) social norms (Mackie et al. 2015). It turns out that the average expectations are not significantly different between the nondisclosure and the identification condition with an average of 6 coins (Table 2), and very similar distributions (Supplementary Materials A.7.1).^7^ The data reveals a positive correlation between expectations and contributions at the individual level (Supplementary Materials A.5.1 and A.7.2), which is in accordance with the concepts of conditional cooperation and reciprocal behavior (Fischbacher et al. 2001; Fischbacher and Gächter 2010).

### Explorative Analysis of Social Ties

Next, I explore the effect of pre-existing social relations between group members. Since the measurement for the number of friends and relatives in one’s group is not the same across both experimental conditions, they are presented in separate regression models (Table 4). Again, the first two models show results for the whole sample of each experimental condition, whereas the third and fourth column focus on the subsamples who correctly answered the control questions for understanding (cqc).

**Table 4:** Effects of social ties between group members (within single experimental conditions)

DV: contributions	**Nondisclosure**	**Identification**	**Nondisclosure (cqc)**	**Identification (cqc)**
	odds-ratios *(p)*	odds-ratios *(p)*	odds-ratios *(p)*	odds-ratios *(p)*
friends (workshop)^1^	1.211		0.935	
	*(0.64)*		*(0.88)*	
family (workshop)^1^	0.884		0.877	
	*(0.72)*		*(0.71)*	
friends (group)		2.104**		2.017
		*(0.04)*		*(0.12)*
family (group)		1.800*		2.279**
		*(0.06)*		*(0.04)*
*‘socioeconomics’*	*yes*	*yes*	*yes*	*yes*
*exclude misunderstood*	*no*	*no*	*yes*	*yes*
N	72	71	61	52

cqc = only subsample of those who correctly answered both control questions for understanding (*n*= 114). Multilevel ordered logit regressions with random intercepts for villages and playing groups. Odds ratios shown for effect sizes. The dependent variable, contributions, is trichotomized into three categories: 0–1 coin (“low”), 2–8 coins (“some”), and 9–10 coins (“much”). P-values are shown in parentheses. Significance levels: * *p* < 0.10, ** *p* < 0.05, *** *p* < 0.01. A more detailed table which shows all coefficients for socioeconomic variables can be found in the Supplementary Materials (A.8)

The regression results reveal that both friends and family members in one’s group have a strong positive effect on contributions in the condition with revealed identities (Table 4). According to the odds ratios, each additional friend increases the likelihood of contributing at a higher category by over 110%, and each additional family member by approximately 80%. No significant effects are observed for the equivalized number of friends or family members in the larger workshop group under the nondisclosure condition. The same general pattern holds in the subsample restricted to participants who correctly answered both control questions, with larger odds ratios, especially for the effect of family members (128%). The effect of friends, however, is no longer statistically significant in this subsample (p = 0.12).

**Fig. 5:**
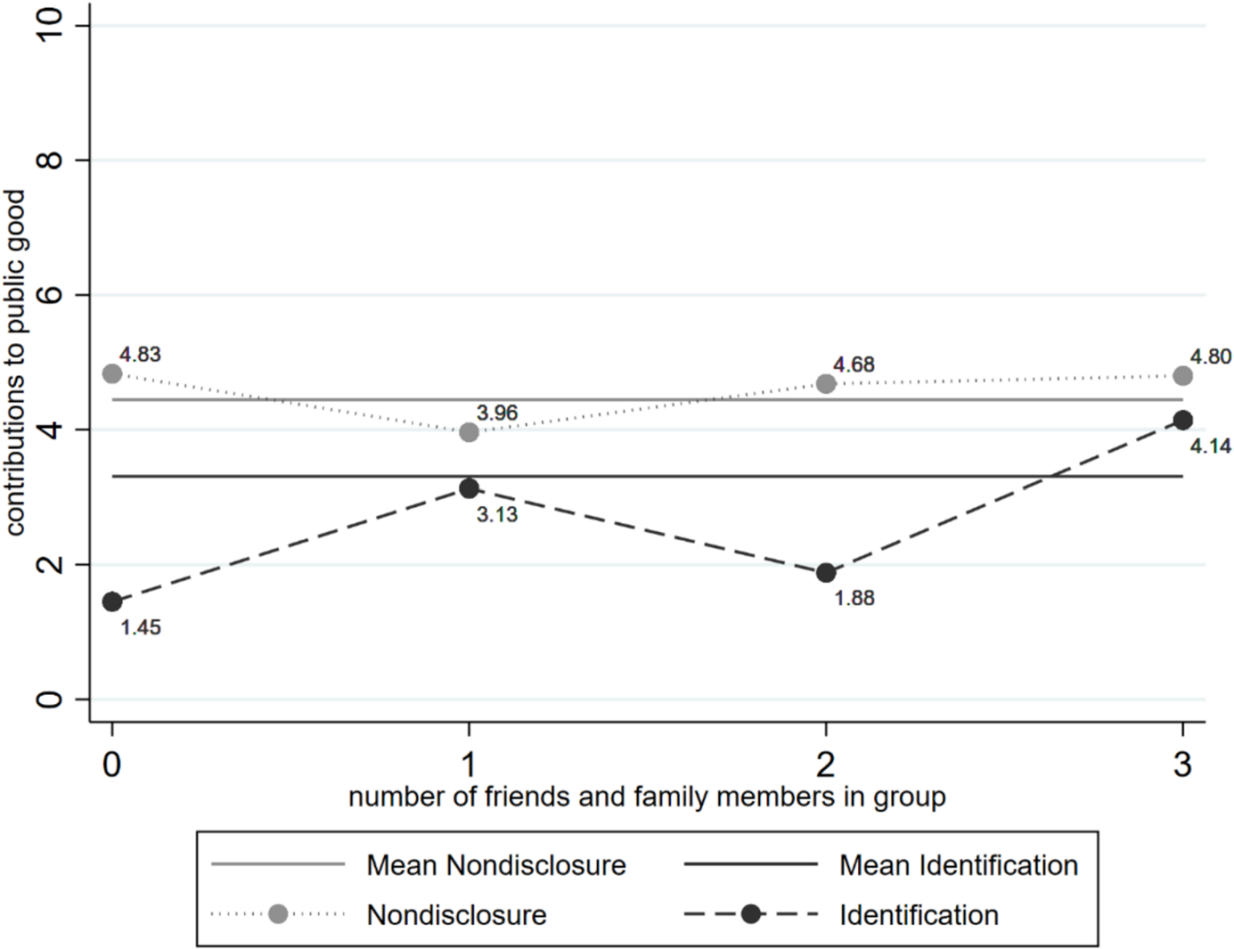
Average contributions for values of social ties across conditions

Figure 5 shows average contributions across both experimental conditions for all values of social ties, measured as the sum of friends and family members. There is an overall positive association between the number of friends and family members in one’s group with contributions in the identification condition. Corresponding, detailed tables can be found in the Supplementary Materials (A.9).^8^ It stands out that with all three group members consisting of friends and family members (Fig. 5),^9^ the average contributions are at about the same level as the overall average contributions in the nondisclosure condition but are lower when fewer friends and family members are present in the group. For the nondisclosure condition, there is no clear association of average contributions with the (equivalent) number of friends and family members in the whole workshop.

## Discussion

This study examined whether the revelation of group members’ identities affects cooperative behavior. An artefactual public good field experiment compared two conditions: one in which group members’ identities remained undisclosed and another in which they were revealed. Importantly, individual decisions were not made public at any time in either condition. Results show that contributions were significantly higher in the nondisclosure setting than in the condition with revealed identities. In other words, the effect of disclosing identities on cooperation was negative.

These results may seem unexpected at first, and not in line with prior findings related to in-group favoritism and minimal group effects in particular (e.g., Tajfel et al. 1971; Balliet et al. 2014). I present in the following a number of potential explanations for the observed effect. The data at hand do not allow a definitive identification of the underlying mechanisms but the explanations provide initial guidance for future investigations.

One plausible explanation for more cooperation in the nondisclosure condition is that participants overestimate the likelihood of being matched with socially close partners, such as family members or simply act risk-averse given the mere possibility. This proposition finds support in average contributions being virtually the same in both conditions when all group members in the identified condition were friends or family members, whereas lower cooperation in the identification condition was primarily found in groups that were not as socially close with each other.

More generally, the nondisclosure condition might create an impression of playing in a larger group, making cooperation appear more valuable or beneficial, even though, in the experiment, participants in both conditions were aware that they would only actually be matched with exactly three other players as their group members (c.f. Bonacich et al. 1976). This could even be based on (intuitive) equity considerations: Contributing to a larger group of unidentified group members may create the perception of a more even distribution of benefits. However, this explanation does not seem in line with some established theories, such as the minimal group paradigm, the standard economic prediction on the effect of decreasing marginal-per-capita-returns, as well as the phenomenon of the “identifiable victim effect”, which proposes more concern towards single, identified persons than towards unknown groups (Schelling 1968; Jenni and Loewenstein 1997).

Another explanation, which aligns well with the present findings, is based on the SIDE model (Reicher et al. 1995). It describes that a group identity and associated social norms become more salient if members are perceived as more similar, which can be achieved through anonymity (Turner et al. 1987, Reicher et al. 1995; Lea et al. 2001). In this study’s context, the undisclosed condition might make the common, social identity on village community level more salient, so that one’s undisclosed group members are projected as prototypical villagers and individual behaviors align, through depersonalization, with the community norm, which may be cooperative. Conversely, the revelation of group members may expose and highlight individual identities, including differences between them, such as in age, socioeconomic status or ethnicity, resulting in a decrease in the perception of a common group identity. Group heterogeneity has been linked to lower performance and cooperativeness, even on larger-scales (e.g., Alesina et al. 1999). The perceived identity of being an (anonymous) member of the village community could, in contrast, be more conducive to cooperation than a group identity potentially and spontaneously generated by the random allocation of four individuals to a new group in the experiment (De Cremer and Vugt 1998). I further observe virtually the same average expectations across both experimental conditions. Interpreting expectations as a measurement of social norms, it can be seen as an indicator in favor of the explanation provided by the SIDE model: While average contributions remain below average stated expectations in both conditions,^10^ the difference, at least, is smaller when identities are not revealed, which emphasizes that the mechanism manipulating the outcome may be the salience of or conformity to a group norm rather than a difference in the norm itself.

A complementary explanation for the negative effect of identity disclosure is offered by the “nasty neighbor effect” (Christensen and Radford 2018; Romano et al. 2024). Revealing individual identities within pre-existing social networks may heighten social comparison or rivalry, particularly in competitive environments such as the Kavangos. Prior research has shown that in-group favoritism and in-group competition can coexist (Romano et al. 2024). In the Kavangos, too, villagers both cooperate and compete in the management of communally used land and in the extraction of natural resources. From an evolutionary perspective, individuals within social groups may attempt to balance cooperation – ensuring their group thrives relative to other groups – with competition, aimed at improving one’s own standing within the group (McCallum et al. 1985; Bell et al. 2009; Bowles 2009, Pisor and Ross 2024). This interpretation aligns with effects predicted by the SIDE model: while norms at the broader group level encourage cooperation, interactions with a smaller set of identified individuals may activate more competitive dynamics.

Further results found with this study reveal a strong preference to cooperate with family members as well as with friends. In line with these observations, I find slightly lower within-group variability of contributions, but larger variability across groups in the identification condition than in the nondisclosure condition.

Regarding potential limitations, it should be noted that with 12 players visible in the “nondisclosure” condition, one’s group members were not fully anonymous, faceless, and distant participants, such as in online experiments. It cannot be ruled out that (minimal) group effects may still have emerged because the other 11 workshop participants were visible and pre-existing social relations between them could still influence behavior, even though the actual allocation of groups playing together remained unknown. Future research could explore truly anonymous conditions. To disentangle and confirm or reject explanations discussed above, the role of village-level identities and norms as well as “nasty neighbor” dynamics could be investigated by further varying the composition of the group. For example, participants could be matched with group members from different backgrounds, such as other villages, so that a perception of direct competitors (nasty neighbor effects) or a shared social identity can be directly tested as relevant mechanisms. Another potentially interesting aspect that my study does not address empirically is a distinction between identifiability of oneself in the group and identification of the other group members, as discussed by Reicher et al. (1995) as well as Spears and Postmes (2015). These could be split up to differentiate effects through one-way revelations, which has already been studied in the context of dictator games (Burnham 2003). More broadly, all findings’ as well as their interpretations’ ecological and external validity must be developed through further research with diverse samples, contextual variation, and, ideally, triangulation across multiple study methods (Pisor and Ross 2024).

## Conclusion

Contrary to what several theories presented in Sect. "Introduction" suggest, making group members identifiable in social dilemma situations does, according to my findings, not appear to promote cooperation. On the contrary, it was observed that groups of nondisclosed individuals act more cooperatively. This can be explained by different, not necessarily mutually exclusive mechanisms, ranging from misperceptions of the likelihood of being matched with socially close group members, or risk-aversion anticipating such possibilities, to in-group competition (“nasty neighbor effects”) and predictions made by the SIDE model (Reicher et al. 1995).

Regarding the SIDE model, it is important to note that anonymity or depersonalization effects are not general but context-dependent, varying with the individuals involved, their social identity and the associated social norms (Lea et al. 2001). When salient group norms are antisocial or destructive, increased group identity achieved through anonymity can produce undesirable outcomes, consistent with classical theories of group dynamics (Le Bon 1896; Festinger et al. 1952; Zimbardo 1969). Consequently, drawing concrete policy implications to foster cooperation is challenging, as the direction of these effects may remain unpredictable. This inherent uncertainty, combined with evidence from history, cautions against deliberate attempts to instrumentalize group dynamics or depersonalization effects.

More broadly, the identifiability of individuals and their behavior will in most contexts depend on the specific dilemma and its setting, rather than being malleable through interventions. Still, findings on identification effects are relevant, for example, to the discussion about group size and cooperation, with anonymity working as an additional mechanism, next to the marginal-per-capita-return, through which variations in group size could affect cooperative behavior.^11^ Perceptions of anonymity likely increase with group size as each individual’s behavior will not only make less of a relative difference for the group outcome but will also receive less attention in larger as compared to smaller groups.

Further insights found by this study include an empirically observed positive association between social closeness and cooperation. This applies both to kinship and to non-kin relationships, such as friendship.

Finally, the results show that, although identity disclosure did not increase the overall variation in contribution decisions, it was associated with lower within-group and higher between-group heterogeneity. In other words, contributions were more similar within groups and more distinct between groups compared to the nondisclosure condition, a finding of methodical relevance for future experimental studies.

## Supplementary Information

Below is the link to the electronic supplementary material.

Supplementary file1 (DOCX 1053 KB)

## Data Availability

Data and analysis code are available in Zenodo, a public repository: https://zenodo.org/records/17014730

## Footnotes

1 It is worth mentioning that two additional experimental conditions were part of the randomization process. These conditions allowed communication between participants and the results have been published in Hoenow and Pourviseh (2024). Since all conditions were randomly allocated but only two conditions could be played per village due limitations in resources, the conditions relevant to this study were not all played in each village. They were played six times each across ten villages.

2 Given that members from the whole workshop are indeed visible, the nondisclosure condition is actually not keeping group members entirely anonymous, but it is the group allocation that remains confidential. I therefore refrain from labeling this as an “anonymous” condition, even though I draw on respective literature for this study.

3 One participant left after playing the public good game so he could not complete the post-experimental survey.

4 Indeed, there was a high variability in contributions across villages (see Supplementary Information A.6.1). According to the F_ST_ statistic, 43% of the total variance in contributions is attributable to differences between villages, with the remaining 57% reflecting within-village variation. This substantial clustering of behavior at the village level underscores the appropriateness of using multilevel models, including random intercepts in the analysis.

5 I based a power calculation on standard deviations found in a public good lab experiment by Fehr and Gächter (2000) and find that at 80% power (1 – 𝛽) and 5% type I error rate (𝛼) a sample size of 72 per condition allows detecting an average 1.5 coins pairwise-difference in contributions in a two-tailed t-test. In my study, I observe standard deviations that are slightly larger as Fehr and Gächter (2000) for the unrestricted sample and an effect size below the assumed 1.5 experimental coins. Therefore, the observed effect is only significant at the 10% level. Focusing on the subsample that correctly answered both control questions though, the difference is larger so that a simple test of means results in a difference significant at the 1% level.

6 Further alternative model specifications include the addition of expectations as an explanatory variable (Supplementary Materials A.5.1), the inclusion of interaction effects between the experimental condition and social ties (Supplementary Materials A.5.2). Across both variations of the main model, effects of the identification condition on contributions remain negative and significant.

7 Regression tables that use expectations as the dependent variable can be found in the Supplementary Materials A.7.3 and A.7.4. They confirm that expectations are not affected by the experimental conditions and show that they do not depend on the group composition, either. See also Supplementary Materials A.7.5 for a table that shows the average expectations for any given number of friends and family members.

8 Notably, the number (and thereby share) of friends and family members in one’s group is larger in the identification condition than what I calculated as equivalent from the larger group of 11 other players for the nondisclosure condition Supplementary Materials (A.2 and A.9).

9 This also holds true when looking at friends and family members separately (see Supplementary Materials A.9). Even in groups that consist entirely of family members, average contributions were not higher than in the nondisclosure condition.

10 This is not an uncommon finding. A tendency to contribute slightly less than one’s group members in order to benefit from their cooperation has been observed and discussed in previous studies (e.g., Fischbacher and Gächter 2010).

11 The direct effects of group size on cooperation have been studied intensively, often with a focus on the difference in the marginal-per-capita-return that comes along with it (Marwell and Ames 1979; Isaac and Walker 1988; Isaac et al. 1994; Nosenzo et al. 2015; Weimann et al. 2019; Wu et al. 2020).

## References

[CR1] Alesina, A., Baqir, R., & Easterly, W. (1999). Public goods and ethnic divisions. *The Quarterly Journal of Economics,**114*(4), 1243–1284. 10.1162/003355399556269

[CR2] Anderies, J. M., Janssen, M. A., Bousquet, F., Cardenas, J. C., Castillo, D., Lopez, M. C., Tobias, R., Vollan, B., & Wutich, A. (2011). The challenge of understanding decisions in experimental studies of common pool resource governance. *Ecological Economics,**70*(9), 1571–1579. 10.1016/j.ecolecon.2011.01.011

[CR3] Andreoni, J., & Petrie, R. (2004). Public goods experiments without confidentiality: A glimpse into fund-raising. *Journal of Public Economics,**88*(7–8), 1605–1623. 10.1016/S0047-2727(03)00040-9

[CR4] Apicella, C. L., Marlowe, F. W., Fowler, J. H., & Christakis, N. A. (2012). Social networks and cooperation in hunter-gatherers. *Nature,**481*(7382), 497–501. 10.1038/nature10736 22281599 10.1038/nature10736 PMC3340565

[CR5] Balliet, D., Wu, J., & De Dreu, C. K. (2014). Ingroup favoritism in cooperation: A meta-analysis. *Psychological Bulletin,**140*(6), 1556. 10.1037/a0037737 25222635 10.1037/a0037737

[CR6] Beheim, B. A., & Bell, A. V. (2024). Why cultural distance can promote–or impede–group-beneficial outcomes. *Evolutionary Human Sciences,**6*, Article e14. 10.1017/ehs.2024.8 38516367 10.1017/ehs.2024.8 PMC10955364

[CR7] Bell, A. V., Richerson, P. J., & McElreath, R. (2009). Culture rather than genes provides greater scope for the evolution of large-scale human prosociality. *Proceedings of the National Academy of Sciences of the United States of America,**106*(42), 17671–17674. 10.1073/pnas.0903232106 19822753 10.1073/pnas.0903232106 PMC2764900

[CR8] Ben-Ner, A., McCall, B. P., Stephane, M., & Wang, H. (2009). Identity and in-group/out-group differentiation in work and giving behaviors: Experimental evidence. *Journal of Economic Behavior & Organization,**72*(1), 153–170. 10.1016/j.jebo.2009.05.007

[CR9] Billig, M., & Tajfel, H. (1973). Social categorization and similarity in intergroup behaviour. *European Journal of Social Psychology,**3*(1), 27–52. 10.1002/ejsp.2420030103

[CR10] Bohnet, I., & Frey, B. S. (1999). The sound of silence in prisoner’s dilemma and dictator games. *Journal of Economic Behavior & Organization,**38*(1), 43–57. 10.1016/S0167-2681(98)00121-8

[CR11] Bonacich, P., Shure, G. H., Kahan, J. P., & Meeker, R. J. (1976). Cooperation and group size in the N-person prisoners’ dilemma. *Journal of Conflict Resolution,**20*(4), 687–706. 10.1177/002200277602000406

[CR12] Bowles, S. (2009). Did warfare among ancestral hunter-gatherers affect the evolution of human social behaviors? *Science,**324*(5932), 1293–1298. 10.1126/science.1168112 19498163 10.1126/science.1168112

[CR13] Brewer, M. B. (1979). In-group bias in the minimal intergroup situation: A cognitive-motivational analysis. *Psychological Bulletin,**86*(2), 307–324. 10.1037/0033-2909.86.2.307

[CR14] Brewer, M. B., & Caporael, L. R. (1990). Selfish genes vs. selfish people: Sociobiology as origin myth. *Motivation and Emotion,**14*, 237–243. 10.1007/BF00996182

[CR15] Brosig, J., & Weimann, J. (2003). The effect of communication media on cooperation. *German Economic Review,**4*(2), 217–241. 10.1111/1468-0475.00080

[CR16] Burnham, T. C. (2003). Engineering altruism: A theoretical and experimental investigation of anonymity and gift giving. *Journal of Economic Behavior & Organization,**50*(1), 133–144. 10.1016/S0167-2681(02)00044-6

[CR17] Butz, B., & Harbring, C. (2021). The effect of disclosing identities in a socially incentivized public good game. *Games,**12*(2), Article 32. 10.3390/g12020032

[CR18] Candelo, N., Eckel, C., & Johnson, C. (2018). Social distance matters in dictator games: Evidence from 11 Mexican villages. *Games,**9*(4), Article 77. 10.3390/g9040077

[CR19] Chang, J. (2008). The role of anonymity in deindividuated behavior: A comparison of deindividuation theory and the social identity model of deindividuation effect. *The Pulse,**6*(1), 1–8.

[CR20] Christensen, C., & Radford, A. N. (2018). Dear enemies or nasty neighbors? Causes and consequences of variation in the responses of group-living species to territorial intrusions. *Behavioral Ecology,**29*(5), 1004–1013. 10.1093/beheco/ary010

[CR21] Darley, J. M., & Latané, B. (1968). Bystander intervention in emergencies: Diffusion of responsibility. *Journal of Personality and Social Psychology,**8*(4, Pt.1), 377–383. 10.1037/h0025589 5645600 10.1037/h0025589

[CR22] Darwin, C. R. (1859). *On the origin of species by means of natural selection, or the preservation of favoured races in the struggle for life*. John Murray. PMC5184128 30164232

[CR23] Dawes, R. M., McTavish, J., & Shaklee, H. (1977). Behavior, communication, and assumptions about other people’s behavior in a commons dilemma situation. *Journal of Personality and Social Psychology,**35*(1), 1–11. 10.1037/0022-3514.35.1.1

[CR24] De Cremer, D., & Van Vugt, M. (1998). Collective Identity and Cooperation in a Public Goods Dilemma: A Matter of Trust or Self-Efficacy. *Current Research in Social Psychology,**3*(1), 1–11.

[CR25] Dubrovsky, V. J., Kiesler, S., & Sethna, B. N. (1991). The equalization phenomenon: Status effects in computer-mediated and face-to-face decision-making groups. *Human-Computer Interaction,**6*(2), 119–146. 10.1207/s15327051hci0602_2

[CR26] Eckel, C. C., Wilson, R. K., Scharlemann, J., & Kacelnik, A. (2001). The value of a smile: Game theory with a human face. *Journal of Economic Psychology*. 10.1016/S0167-4870(01)00059-9

[CR27] Fehr, E., & Gächter, S. (2000). Cooperation and punishment in public goods experiments. *American Economic Review,**90*(4), 980–994. 10.1257/aer.90.4.980

[CR28] Festinger, L., Pepitone, A., & Newcomb, T. (1952). Some consequences of de-individuation in a group. *The Journal of Abnormal and Social Psychology,**47*(2S), 382–389. 10.1037/h0057906 10.1037/h0057906 14937978

[CR29] Fischbacher, U., & Gächter, S. (2010). Social preferences, beliefs, and the dynamics of free riding in public goods experiments. *American Economic Review,**100*(1), 541–556. 10.1257/aer.100.1.541

[CR30] Fischbacher, U., Gächter, S., & Fehr, E. (2001). Are people conditionally cooperative? Evidence from a public goods experiment. *Economics Letters,**71*(3), 397–404. 10.1016/S0165-1765(01)00394-9

[CR31] Fox, J., & Guyer, M. (1977). Group size and others’ strategy in an N-person game. *Journal of Conflict Resolution,**21*(2), 323–338. 10.1177/002200277702100206

[CR32] Fox, J., & Guyer, M. (1978). “Public” Choice and Cooperation in n-Person Prisoner’s Dilemma. *Journal of Conflict Resolution,**22*(3), 469–481. 10.1177/002200277802200307

[CR33] Genevsky, A., Västfjäll, D., Slovic, P., & Knutson, B. (2013). Neural Underpinnings of the Identifiable Victim Effect: Affect Shifts Preferences for Giving. *Journal of Neuroscience,**33*(43), 17188–17196. 10.1523/JNEUROSCI.2348-13.2013 24155323 10.1523/JNEUROSCI.2348-13.2013 PMC3807035

[CR34] Ghate, R., Ghate, S., & Ostrom, E. (2013). Cultural norms, cooperation, and communication: Taking experiments to the field in indigenous communities. *International Journal of the Commons,**7*(2), 498–520.

[CR35] Gintis, H., Bowles, S., Boyd, R., & Fehr, E. (2003). Explaining altruistic behavior in humans. *Evolution and Human Behavior,**24*(3), 153–172. 10.1016/S1090-5138(02)00157-5

[CR36] Goette, L., Huffman, D., & Meier, S. (2006). The impact of group membership on cooperation and norm enforcement: Evidence using random assignment to real social groups. *American Economic Review,**96*(2), 212–216. 10.1257/000282806777211658

[CR37] Hamilton, W. D. (1964). The genetical evolution of social behaviour. Part II. *Journal of Theoretical Biology,**7*(1), 17–52. 10.1016/0022-5193(64)90039-6 5875340 10.1016/0022-5193(64)90039-6

[CR38] Handley, C., & Mathew, S. (2020). Human large-scale cooperation as a product of competition between cultural groups. *Nature Communications,**11*(1), Article 702. 10.1038/s41467-020-14416-8 10.1038/s41467-020-14416-8 PMC7000669 32019930

[CR39] Henrich, J., & Henrich, N. (2006). Culture, evolution and the puzzle of human cooperation. *Cognitive Systems Research,**7*(2–3), 220–245. 10.1016/j.cogsys.2005.11.010

[CR40] Hinz, M. O. (2003). *Without *chiefs* there would be no game: customary law and nature conservation.* Out of Africa Publishers.

[CR41] Hoenow, N. C., & Pourviseh, A. (2024). Intragroup communication in social dilemmas: An artefactual public good field experiment in small-scale communities. *Judgment and Decision Making,**19*, Article e1. 10.1017/jdm.2023.38

[CR42] Isaac, R. M., & Walker, J. M. (1988). Group size effects in public goods provision: The voluntary contributions mechanism. *The Quarterly Journal of Economics,**103*(1), 179–199. 10.2307/1882648

[CR43] Isaac, R. M., Walker, J. M., & Williams, A. W. (1994). Group size and the voluntary provision of public goods: Experimental evidence utilizing large groups. *Journal of Public Economics,**54*(1), 1–36. 10.1016/0047-2727(94)90068-X

[CR44] Jenni, K., & Loewenstein, G. (1997). Explaining the identifiable victim effect. *Journal of Risk and Uncertainty,**14*, 235–257. 10.1023/A:1007740225484

[CR45] Kramer, R. M. (1989). *When the going gets tough: The effects of resource scarcity on group conflict and cooperation*. Stanford University.

[CR46] Latané, B., & Dabbs, J. M., Jr. (1975). Sex, Group Size and Helping in Three Cities. *Sociometry,**38*(2), 180–194. https://www.jstor.org/stable/2786599.

[CR47] Latané, B., & Nida, S. (1981). Ten years of research on group size and helping. *Psychological Bulletin,**89*(2), 308–324. 10.1037/0033-2909.89.2.308

[CR48] Le Bon, G. (1896). *Psychologie des foules.* F. Alcan.

[CR49] Lea, M., Spears, R., & De Groot, D. (2001). Knowing me, knowing you: Anonymity effects on social identity processes within groups. *Personality and Social Psychology Bulletin,**27*(5), 526–537. 10.1177/0146167201275002

[CR50] Mackie, G., Moneti, F., Shakya, H., & Denny, E. (2015). What are social norms? How are they measured. *University of California at San Diego-UNICEF Working Paper, San Diego*.

[CR51] Majdandžić, J., Bauer, H., Windischberger, C., Moser, E., Engl, E., & Lamm, C. (2012). The human factor: Behavioral and neural correlates of humanized perception in moral decision making. *PLoS ONE,**7*(10), Article e47698. 10.1371/journal.pone.0047698 23082194 10.1371/journal.pone.0047698 PMC3474750

[CR52] Mann, L., Newton, J. W., & Innes, J. M. (1982). A test between deindividuation and emergent norm theories of crowd aggression. *Journal of Personality and Social Psychology,**42*(2), 260–272. 10.1037/0022-3514.42.2.260

[CR53] Marwell, G., & Ames, R. E. (1979). Experiments on the provision of public goods. I. Resources, interest, group size, and the free-rider problem. *American Journal of Sociology,**84*(6), 1335–1360. 10.1086/226937

[CR54] McCallum, D. M., Harring, K., Gilmore, R., Drenan, S., Chase, J. P., Insko, C. A., & Thibaut, J. (1985). Competition and cooperation between groups and between individuals. *Journal of Experimental Social Psychology,**21*(4), 301–320. 10.1016/0022-1031(85)90032-0

[CR55] Namibia Statistics Agency. (2023a). *Kavango West region*. https://nsa.org.na/census/kavango-west-region Accessed: August 2025

[CR56] Namibia Statistics Agency. (2023b). *Kavango East region*. https://nsa.org.na/census/kavango-east-region/ Accessed: August 2025

[CR57] Namibian Ministry of Lands and Resettlements (2015). Integrated Regional Land Use Plan for the Kavango East Region Baseline Report (Volume 1). Ministry of Lands and Resettlements, Government of Namibia. http://www.mlr.gov.na/documents/20541/497602/Kavango+East+Volume+1+March+2015.pdf/911cdfb1-bc85-4e8f-ad20-7895b7c35244

[CR58] Nosenzo, D., Quercia, S., & Sefton, M. (2015). Cooperation in small groups: The effect of group size. *Experimental Economics,**18*, 4–14. 10.1007/s10683-013-9382-8

[CR59] Peters, H. E., Ünür, A. S., Clark, J., & Schulze, W. D. (2004). Free-riding and the provision of public goods in the family: A laboratory experiment*. *International Economic Review,**45*(1), 283–299. 10.1111/j.1468-2354.2004.00126.x

[CR60] Pisor, A. C., & Ross, C. T. (2024). Parochial altruism: What it is and why it varies. *Evolution and Human Behavior,**45*(1), 2–12. 10.1016/j.evolhumbehav.2023.06.005

[CR61] Pröpper, Michael, Gröngröft, A., Finckh, M., Stirn, S., De Cauwer, V., Fernanda, L., Masamba, W., Murray-Hudson, M., Schmidt, L., Strohbach, B., & Jürgens, N. (2015). *The Future Okavango—Findings, Scenarios, and Recommendations for Action.* University of Hamburg – Biocentre Klein Flottbek. https://www.future-okavango.org/downloads/TFO_Report_engl_compiled_small_version.pdf Accessed: August 2025

[CR62] Rege, M., & Telle, K. (2004). The impact of social approval and framing on cooperation in public good situations. *Journal of Public Economics,**88*(7–8), 1625–1644. 10.1016/S0047-2727(03)00021-5

[CR63] Reicher, S. D., Spears, R., & Postmes, T. (1995). A social identity model of deindividuation phenomena. *European Review of Social Psychology,**6*(1), 161–198. 10.1080/14792779443000049

[CR64] Romano, A., Gross, J., & De Dreu, C. K. (2024). The nasty neighbor effect in humans. *Science Advances,**10*(26), Article eadm7968. 10.1126/sciadv.adm7968 38924403 10.1126/sciadv.adm7968 PMC11204206

[CR65] Roth, A. (1995). Bargaining experiments. *Handbook of Experimental Economics*, 253–348.

[CR66] Sampaio, W. M., Freitas, A. L., Rêgo, G. G., Morello, L. Y., & Boggio, P. S. (2023). Effects of co-players’ identity and reputation in the public goods game. *Scientific Reports,**13*(1), Article 13520. 10.1038/s41598-023-40730-4 37598241 10.1038/s41598-023-40730-4 PMC10439960

[CR67] Savikhin Samek, A., & Sheremeta, R. M. (2014). Recognizing contributors: An experiment on public goods. *Experimental Economics,**17*, 673–690. 10.1007/s10683-013-9389-1

[CR68] Schelling, T. C. (1968). The life you save may be your own. *Problems in Public Expenditure*, 127–162.

[CR69] Smith, K. M., Larroucau, T., Mabulla, I. A., & Apicella, C. L. (2018). Hunter-gatherers maintain assortativity in cooperation despite high levels of residential change and mixing. *Current Biology,**28*(19), 3152–3157. 10.1016/j.cub.2018.07.064 30245106 10.1016/j.cub.2018.07.064

[CR70] Spears, R., & Postmes, T. (2015). Group Identity, Social Influence, and Collective Action Online: Extensions and Applications of the SIDE Model. *The Handbook of the Psychology of Communication Technology*, 23–46. 10.1002/9781118426456.ch2

[CR71] Tajfel, H. (1974). Social identity and intergroup behaviour. *Social Science Information,**13*(2), 65–93. 10.1177/053901847401300204

[CR72] Tajfel, H., & Turner, J. C. (2004). The Social Identity Theory of Intergroup Behavior. In *Political Psychology* (pp. 276–293). Psychology Press. 10.4324/9780203505984-16

[CR73] Tajfel, H., Billig, M. G., Bundy, R. P., & Flament, C. (1971). Social categorization and intergroup behaviour. *European Journal of Social Psychology,**1*(2), 149–178. 10.1002/ejsp.2420010202

[CR74] Turner, J. C., Brown, R. J., & Tajfel, H. (1979). Social comparison and group interest in ingroup favouritism. *European Journal of Social Psychology,**9*(2), 187–204. 10.1002/ejsp.2420090207

[CR75] Turner, J. C., Hogg, M. A., Oakes, P. J., Reicher, S. D., & Wetherell, M. S. (1987). *Rediscovering the social group: A self-categorization theory*. Basil Blackwell. https://psycnet.apa.org/record/1987-98657-000

[CR76] Weimann, J., Brosig-Koch, J., Heinrich, T., Hennig-Schmidt, H., & Keser, C. (2019). Public good provision by large groups–The logic of collective action revisited. *European Economic Review,**118*, 348–363. 10.1016/j.euroecorev.2019.05.019

[CR77] Wichman, H. (1970). Effects of isolation and communication on cooperation in a two-person game. *Journal of Personality and Social Psychology,**16*(1), 114–120. 10.1037/h0029845

[CR78] Wilson, D. S., & Wilson, E. O. (2007). Rethinking the theoretical foundation of sociobiology. *The Quarterly Review of Biology,**82*(4), 327–348. 10.1086/522809 18217526 10.1086/522809

[CR79] Wu, J., Balliet, D., Peperkoorn, L. S., Romano, A., & Van Lange, P. A. M. (2020). Cooperation in groups of different sizes: The effects of punishment and reputation-based partner choice. *Frontiers in Psychology,**10*, Article 2956. 10.3389/fpsyg.2019.02956 32038365 10.3389/fpsyg.2019.02956 PMC6985556

[CR80] Zimbardo, P. G. (1969). The human choice: Individuation, reason, and order versus deindividuation, impulse, and chaos. In Nebraska* Symposium on Motivation*. *17*, 237–307. https://psycnet.apa.org/record/1971-08069-001

