# Supplementary material for: Disclosing Group Members’ Identities Reduces Cooperation in an Artefactual Public Goods Field Experiment
Source: Hum Nat. 2025 Nov 13;36(3):337–59. doi: 10.1007/s12110-025-09508-7 (PMC12644118; doi:10.1007/s12110-025-09508-7)

**Supplementary Materials**

*for*

**Disclosing Group Members’ Identities Reduces Cooperation
in an Artefactual Public Goods Field Experiment**

Nils Christian Hoenow^1,2,3,4^

**^1^** RWI-Leibniz Institute for Economic Research, Hohenzollernstr. 1-3, D-45128 Essen, Germany

**^2^** School of Business and Economics, University of Marburg, Am Plan 2, D-35032 Marburg, Germany

**^3^** Corresponding author: christian.hoenow@rwi-essen.de

**^4^** Declarations of interest: none

August 2025

**A. Appendix**

A.1 The public good game

A.2 Comparison of sample characteristics across experimental conditions

A.3 Full multilevel ordered logit regression model (main specification)

A.4 Multilevel ordered logit regression with random slopes

A.5 Alternative regression model specifications

A.6 Analysis of variability

A.7 Analysis of expectations (beliefs)

A.8 Full models within the experimental conditions (friends and family analysis)

A.9 Average contributions by number of friends and family members

**B. Experimental Protocols**

B.1 Village meeting

B.2 General instructions

B.3 Game instructions public good game

B.4 Poster

B.5 Examples

B.6 Control questions for public use

B.7 Experimental conditions

B.8 Decision making

B.9 Private control questions and expectations

**C. Survey Questions**

**Supplement A: Appendix**

***A.1: The public good game***

The public good game payoffs **P_i_** can be formalized with the following equation for individuals (equation 1) and for the group as a whole **S** (equation 2):

Equation 1:

$$\boldsymbol{P}_{\boldsymbol{i}}\boldsymbol{(}\boldsymbol{e}_{\boldsymbol{i}}\boldsymbol{, c}_{\boldsymbol{i}}\boldsymbol{,}\boldsymbol{c}_{\boldsymbol{j}}\boldsymbol{)=}\boldsymbol{e}_{\boldsymbol{i}}\boldsymbol{- c}_{\boldsymbol{i}}{\boldsymbol{+}\frac{\boldsymbol{m}}{\boldsymbol{n}}\boldsymbol{c}}_{\boldsymbol{i}}\boldsymbol{+}\frac{\boldsymbol{m}}{\boldsymbol{n}}\sum_{\boldsymbol{j=1}}^{\boldsymbol{n-1}} \boldsymbol{c}_{\boldsymbol{j}}$$

Equation 2:

$$\boldsymbol{S}\left( \boldsymbol{e}_{\boldsymbol{i}}\boldsymbol{, c}_{\boldsymbol{i}} \right)\boldsymbol{=}\sum_{\boldsymbol{i=1}}^{\boldsymbol{n}} \boldsymbol{P}_{\boldsymbol{i}}\boldsymbol{(}\boldsymbol{e}_{\boldsymbol{i}}\boldsymbol{, c}_{\boldsymbol{i}}\boldsymbol{)}$$

$$\boldsymbol{S}\left( \boldsymbol{e}_{\boldsymbol{i}}\boldsymbol{, c}_{\boldsymbol{i}} \right)\boldsymbol{=}\boldsymbol{n*P}_{\boldsymbol{i}}\boldsymbol{(}\boldsymbol{e}_{\boldsymbol{i}}\boldsymbol{, c}_{\boldsymbol{i}}\boldsymbol{)}$$

$$\boldsymbol{S}\left( \boldsymbol{e}_{\boldsymbol{i}}\boldsymbol{, c}_{\boldsymbol{i}} \right)\boldsymbol{=}\boldsymbol{n*e}_{\boldsymbol{i}}\boldsymbol{- n*c}_{\boldsymbol{i}}\boldsymbol{+m}\sum_{\boldsymbol{i=1}}^{\boldsymbol{n}} \boldsymbol{c}_{\boldsymbol{i}}$$

P_i_ = payoff of player i

e_i_ = endowment player i

c_i_ = contribution decision of player i,  **∈**{ 0 ,… 10}

c_j_ = contribution decision of player j,  **∈**{ 0 ,… 10}

m = multiplier of group account

n = number of players in the group

S = sum of all players’ payoffs

With an endowment (e) of 10 coins, a group size (n) of 4 player and the social multiplier (m) set to 2, the payoff equation becomes (equation 3):

Equation 3:

$$\boldsymbol{P}_{\boldsymbol{i}}\boldsymbol{(}{\boldsymbol{c}_{\boldsymbol{i}}\boldsymbol{, c}}_{\boldsymbol{j}}\boldsymbol{)=10}\boldsymbol{- c}_{\boldsymbol{i}}{\boldsymbol{+}\frac{\boldsymbol{2}}{\boldsymbol{4}}\boldsymbol{c}}_{\boldsymbol{i}}\boldsymbol{+}\frac{\boldsymbol{2}}{\boldsymbol{4}}\sum_{\boldsymbol{j=1}}^{\boldsymbol{3}} \boldsymbol{c}_{\boldsymbol{j}}$$

$$\boldsymbol{P}_{\boldsymbol{i}}\boldsymbol{(}{\boldsymbol{c}_{\boldsymbol{i}}\boldsymbol{, c}}_{\boldsymbol{j}}\boldsymbol{)=10}{\boldsymbol{-}\frac{\boldsymbol{1}}{\boldsymbol{2}}\boldsymbol{c}}_{\boldsymbol{i}}\boldsymbol{+}\frac{\boldsymbol{1}}{\boldsymbol{2}}\sum_{\boldsymbol{j=1}}^{\boldsymbol{3}} \boldsymbol{c}_{\boldsymbol{j}}$$

With c_i_ being the only variable that player *i* can manipulate, it becomes obvious that contributing nothing is the individually best option. For the group payoff (equation 4), on the other hand, there is:

Equation 4:

$$\boldsymbol{S}\left( \boldsymbol{c}_{\boldsymbol{i}} \right)\boldsymbol{=}\boldsymbol{4*P}_{\boldsymbol{i}}\boldsymbol{(}\boldsymbol{c}_{\boldsymbol{i}}\boldsymbol{)}$$

$$\boldsymbol{S}\left( \boldsymbol{c}_{\boldsymbol{i}} \right)\boldsymbol{= 40}\boldsymbol{- 4*c}_{\boldsymbol{i}}\boldsymbol{+2}\sum_{\boldsymbol{i=1}}^{\boldsymbol{4}} \boldsymbol{c}_{\boldsymbol{i}}$$

$$\boldsymbol{S}\left( \boldsymbol{c}_{\boldsymbol{i}} \right)\boldsymbol{=40}\boldsymbol{- 4*c}_{\boldsymbol{i}}\boldsymbol{+2*4*}\boldsymbol{c}_{\boldsymbol{i}}$$

$$\boldsymbol{S}\left( \boldsymbol{c}_{\boldsymbol{i}} \right)\boldsymbol{=40+4}\boldsymbol{c}_{\boldsymbol{i}}$$

This shows that, for the group, contributing as much as possible leads to the highest payoff. Since c_i_ is capped at 10, this is the social optimum in the game. The minimum payoff in the experiment is 5 coins (25N$) for someone, who contributes everything while in a group with three free riders. The maximum payoff is 25 coins (125N$) for a free rider in a group with three cooperators. Plus, the bonus for correctly estimating the other players’ contribution, the maximum amount that can be earned is 125 + 20 = 145N$.

***A.2*** ***Comparison of sample characteristics across experimental conditions***

| **experimental** | **1** | **2** | **Δ** | **test** |
| --- | --- | --- | --- | --- |
| **condition:** | **nondisclosure** | **identification** | difference | for equality |
|  | Mean (std. dev.) | Mean (std. dev.) | means (std. err.) | p-value |
| contribution | 4.44 (3.67) | 3.31 (3.66) | -1.14 (0.61) | 0.065* |
| contributions^cqc^ | 4.70 (3.70) | 2.92 (3.51) | - 1.78 (0.68) | 0.009*** |
| expectation | 6.01 (2.89) | 5.96 (2.80) | -0.06 (0.47) | 0.907 |
| expectation^cqc^ | 6.10 (2.89) | 5.79 (2.80) | -0.31 (0.53) | 0.568 |
| control q. wrong | 0.15 (-) | 0.26 (-) | 0.11 | 0.150 |
| SD (contribution) | 3.67 (-) | 3.66 (-) | -0.01 | 0.984 |
| SD (contribution) ^cqc^ | 3.70 (-) | 3.51 (-) | -0.20 | 0.692 |
| SD (expectation) | 2.89 (-) | 2.80 (-) | -0.09 | 0.780 |
| SD (expectation) ^cqc^ | 2.89 (-) | 2.80 (-) | -0.08 | 0.837 |
|  |  |  |  |  |
| friends^1^ | [0.64 (0.67)] | 0.77 (1.07) | [0.13 (0.15)] | [0.368] |
| family^1^ | [0.77 (0.76)] | 1.42 (1.25) | [0.65 (1.73)] | [0.000***] |
| age | 34.83 (13.00) | 36.89 (15.27) | 2.05 (2.37) | 0.387 |
| female | 0.56 (-) | 0.51 (-) | -0.05 | 0.738 |
| schooling years | 6.47 (3.36) | 6.70 (3.75) | 0.23 (0.60) | 0.697 |
| social ladder | 2.71 (1.92) | 2.25 (1.95) | -0.45 (0.32) | 0.163 |
| hectares (log) | 1.35 (0.58) | 1.06 (0.51) | -0.29 (0.91) | 0.002*** |
| cattle (log) | 1.65 (1.50) | 1.19 (1.42) | -0.47 (0.24) | 0.059* |
| migrant | 0.01 (-) | 0.10 (-) | 0.08 | 0.063* |
|  |  |  |  |  |
| observations | 72 | 72 |  |  |

p-value for two-tailed t-test or Fisher’s-exact-test in case variable is dichotomous, significance levels: * p<0.10, ** p<0.05, *** p<0.01

cqc = only subsample of those who correctly answered both control questions for understanding (n = 114)

SD = standard deviation

^1^ While for the identification condition the variables for friends and family members were elicited on group level, groups were not
 revealed in the nondisclosure condition, and I therefore asked for the number of friends and family members and the whole workshop. In
 order to make the measurement in the nondisclosure condition more comparable, I divide by the number of other workshop participants
 (11) and multiply by group size minus one player (3).

***A.3: Full multilevel ordered logit regression model*** *(main specification)*

| DV: contributions | **model 1** | **model 2** | **model 3** | **model 4** |
| --- | --- | --- | --- | --- |
|  | coefficient *(p)* | coefficient *(p)* | coefficient *(p)* | coefficient *(p)* |
|  |  |  |  |  |
| 2: Identification | -1.199* | -1.314** | -1.594** | -1.623** |
|  | *(0.05)* | *(0.03)* | *(0.02)* | *(0.01)* |
| age |  | 0.019 |  | 0.024 |
|  |  | *(0.23)* |  | *(0.17)* |
| female (d) |  | 0.042 |  | -0.302 |
|  |  | *(0.91)* |  | *(0.49)* |
| schooling years |  | -0.081 |  | -0.011 |
|  |  | *(0.20)* |  | *(0.88)* |
| social ladder (0-10) |  | -0.038 |  | 0.064 |
|  |  | *(0.71)* |  | *(0.54)* |
| hectares (log) |  | -0.277 |  | -0.056 |
|  |  | *(0.44)* |  | *(0.90)* |
| cattle owned (log) |  | 0.067 |  | -0.023 |
|  |  | *(0.62)* |  | *(0.88)* |
| migrant (d) |  | 1.095 |  | 0.877 |
|  |  | *(0.16)* |  | *(0.33)* |
| Thresholds |  |  |  |  |
| low → some | -0.977** | -1.186 | -1.049** | -0.211 |
|  | *(0.04)* | *(0.27)* | *(0.03)* | *(0.86)* |
| some → much | 1.324*** | 1.250 | 1.121** | 2.051* |
|  | *(0.01)* | *(0.25)* | *(0.02)* | *(0.09)* |
| Random variances |  |  |  |  |
| cons(village) | 0.700 | 0.675 | 0.831 | 0.791 |
|  | *(0.32)* | *(0.33)* | *(0.33)* | *(0.33)* |
| cons(groups) | 0.760 | 0.780 | 0.189 | 0.223 |
|  | *(0.21)* | *(0.24)* | *(0.70)* | *(0.67)* |
| N | 144 | 143 | 114 | 113 |

*Multilevel ordered logit regressions with random intercepts for villages and playing groups. Reported coefficients are log-odds (logit) estimates. The dependent variable, contributions, is trichotomized into three categories: 0–1 coin (“low”), 2–8 coins (“some”), and 9–10 coins (“much”). Threshold coefficients (“low → some” and “some → much”) represent the estimated cutoff points between adjacent categories on the latent contribution scale. Random-effect variances indicate between-village and between-group heterogeneity (nested within villages) in the intercepts. Models 1 and 2 show results for the whole sample, whereas models 3 and 4 focus on participants who have correctly answered both control questions for understanding. P-values are shown in parentheses. Significance levels: * p < 0.10, ** p < 0.05, *** p < 0.01.*

***A.4:*** ***Multilevel ordered logit regression with random slopes***

| DV: contributions | **model 1** | **model 2** | **model 3** | **model 4** |
| --- | --- | --- | --- | --- |
|  | coefficient *(p)* | coefficient *(p)* | coefficient *(p)* | coefficient *(p)* |
|  |  |  |  |  |
| T: Identification | -1.034 | -1.208* | -1.412** | -1.533** |
|  | *(0.12)* | *(0.27)* | *(0.05)* | *(0.03)* |
| age |  | 0.019 |  | 0.024 |
|  |  | *(0.24)* |  | *(0.18)* |
| female (d) |  | 0.013 |  | -0.303 |
|  |  | *(0.97)* |  | *(0.50)* |
| schooling years |  | -0.082 |  | 0.012 |
|  |  | *(0.20)* |  | *(0.87)* |
| social ladder (0-10) |  | -0.042 |  | 0.062 |
|  |  | *(0.69)* |  | *(0.56)* |
| hectares (log) |  | -0.271 |  | -0.058 |
|  |  | *(0.45)* |  | *(0.89)* |
| cattle owned (log) |  | 0.072 |  | -0.021 |
|  |  | *(0.60)* |  | *(0.89)* |
| migrant (d) |  | 1.035 |  | 0.819 |
|  |  | *(0.19)* |  | *(0.36)* |
| Thresholds |  |  |  |  |
| low → some | -0.880** | -1.166 | -0.919** | -0.166 |
|  | *(0.03)* | *(0.27)* | *(0.01)* | *(0.88)* |
| some → much | 1.415*** | 1.266 | 1.227*** | 2.079* |
|  | *(0.00)* | *(0.23)* | *(0.00)* | *(0.07)* |
| Random variances |  |  |  |  |
| slope(village) | 0.892 | 0.752 | 1.407 | 1.161 |
|  | *(0.44)* | *(0.51)* | *(0.39)* | *(0.46)* |
| cons(village) | 0.276 | 0.324 | 0.354 | 0.339 |
|  | *(0.60)* | *(0.58)* | *(0.48)* | *(0.54)* |
| cons(group) | 0.722 | 0.748 | 0.077 | 0.183 |
|  | *(0.23)* | *(0.26)* | *(0.86)* | *(0.72)* |
| N | 144 | 143 | 114 | 113 |

*Multilevel ordered logit regressions with random intercepts for villages and playing groups and random slopes for villages. Reported coefficients are log-odds (logit) estimates. The dependent variable, contributions, is trichotomized into three categories: 0–1 coin (“low”), 2–8 coins (“some”), and 9–10 coins (“much”). Threshold coefficients (“low → some” and “some → much”) represent the estimated cutoff points between adjacent categories on the latent contribution scale. Random-effect variances indicate between-village and between-group heterogeneity (nested within villages) in the intercepts and variation in the treatment effect across villages. Models 1 and 2 show results for the whole sample, whereas models 3 and 4 focus on participants who have correctly answered both control questions for understanding. P-values are shown in parentheses. Significance levels: * p < 0.10, ** p < 0.05, *** p < 0.01.*

***A.*5: Alternative regression model specifications**

***A.5.1:*** Alternative model specification: expectations added as explanatory variable

| DV: contributions | **model 1** | **model 2** | **model 3** | **model 4** |
| --- | --- | --- | --- | --- |
|  | coefficient *(p)* | coefficient *(p)* | coefficient *(p)* | coefficient *(p)* |
|  |  |  |  |  |
| T: Identification | -1.182* | -1.376** | -1.578** | -1.681*** |
|  | *(0.05)* | *(0.02)* | *(0.01)* | *(0.01)* |
| expectation | 0.172** | 0.195*** | 0.206*** | 0.208** |
|  | *(0.02)* | *(0.01)* | *(0.01)* | *(0.01)* |
| age |  | 0.023 |  | 0.026 |
|  |  | *(0.15)* |  | *(0.14)* |
| female (d) |  | 0.174 |  | -0.080 |
|  |  | *(0.66)* |  | *(0.85)* |
| schooling years |  | -0.064 |  | 0.028 |
|  |  | *(0.32)* |  | *(0.70)* |
| social ladder (0-10) |  | -0.057 |  | 0.043 |
|  |  | *(0.59)* |  | *(0.68)* |
| hectares (log) |  | -0.377 |  | -0.131 |
|  |  | *(0.29)* |  | *(0.75)* |
| cattle owned (log) |  | 0.090 |  | -0.005 |
|  |  | *(0.51)* |  | *(0.97)* |
| migrant (d) |  | 1.386* |  | 1.269 |
|  |  | *(0.08)* |  | *(0.16)* |
| Thresholds |  |  |  |  |
| low → some | 0.047 | 0.160 | 0.158 | 1.181 |
|  | *(0.94)* | *(0.89)* | *(0.80)* | *(0.35)* |
| some → much | 2.383*** | 2.650** | 2.394*** | 3.511*** |
|  | *(0.00)* | *(0.03)* | *(0.00)* | *(0.01)* |
| Random variances |  |  |  |  |
| cons(village) | 0.698 | 0.684 | 0.883 | 0.925 |
|  | *(0.29)* | *(0.29)* | *(0.27)* | *(0.27)* |
| cons(groups) | 0.560 | 0.549 | 0.000 | 0.036 |
|  | *(0.29)* | *(0.34)* | *(1.00)* | *(0.93)* |
| N | 144 | 143 | 114 | 113 |

*Multilevel ordered logit regressions with random intercepts for villages and playing groups. Reported coefficients are log-odds (logit) estimates. The dependent variable, contributions, is trichotomized into three categories: 0–1 coin (“low”), 2–8 coins (“some”), and 9–10 coins (“much”). Threshold coefficients (“low → some” and “some → much”) represent the estimated cutoff points between adjacent categories on the latent contribution scale. Random-effect variances indicate between-village and between-group heterogeneity (nested within villages) in the intercepts. Models 1 and 2 show results for the whole sample, whereas models 3 and 4 focus on participants who have correctly answered both control questions for understanding. Adding expectations to the model is possible since it was already shown that expectations do not depend on the experimental conditions. Doing so does not substantially change the main results, i.e., the effect of the identification treatment. P-values are shown in parentheses. Significance levels: * p < 0.10, ** p < 0.05, *** p < 0.01.*

***A.5.2:*** Alternative model specification: interaction term with family and friends added

| DV: contributions | **model 1** | **model 2** | **model 3** | **model 4** |
| --- | --- | --- | --- | --- |
|  | coefficient *(p)* | coefficient *(p)* | coefficient *(p)* | coefficient *(p)* |
|  |  |  |  |  |
| T:Identification | -2.425** | -2.542*** | -3.250*** | -3.314*** |
|  | *(0.01)* | *(0.01)* | *(0.00)* | *(0.00)* |
| FamFds | 0.052 | 0.008 | -0.077 | -0.100 |
|  | *(0.85)* | *(0.98)* | *(0.79)* | *(0.75)* |
| T:Identification#FamFds | 0.522 | 0.542 | 0.776* | 0.778* |
|  | *(0.17)* | *(0.17)* | *(0.07)* | *(0.07)* |
| age |  | 0.015 |  | 0.020 |
|  |  | *(0.35)* |  | *(0.26)* |
| female (d) |  | 0.104 |  | -0.243 |
|  |  | *(0.79)* |  | *(0.59)* |
| schooling years |  | -0.089 |  | 0.013 |
|  |  | *(0.17)* |  | *(0.86)* |
| social ladder (0-10) |  | -0.048 |  | 0.047 |
|  |  | *(0.64)* |  | *(0.65)* |
| hectares (log) |  | -0.254 |  | -0.089 |
|  |  | *(0.48)* |  | *(0.83)* |
| cattle owned (log) |  | 0.089 |  | 0.035 |
|  |  | *(0.52)* |  | *(0.83)* |
| migrant (d) |  | 1.047 |  | 1.041 |
|  |  | *(0.19)* |  | *(0.26)* |
| Thresholds |  |  |  |  |
| low → some | -0.912 | -1.302 | -0.151* | -0.442 |
|  | *(0.14)* | *(0.28)* | *(0.07)* | *(0.74)* |
| some → much | 1.412** | 1.138 | 1.071 | 1.846 |
|  | *(0.03)* | *(0.35)* | *(0.10)* | *(0.18)* |
| Random variances |  |  |  |  |
| cons(village) | 0.830 | 0.720 | 0.894 | 0.846 |
|  | *(0.24)* | *(0.28)* | *(0.28)* | *(0.30)* |
| cons(groups) | 0.534 | 0.564 | 0.091 | 0.084 |
|  | *(0.32)* | *(0.35)* | *(0.84)* | *(0.86)* |
| N | 143 | 143 | 114 | 113 |

*Multilevel ordered logit regressions with random intercepts for villages and playing groups. Reported coefficients are log-odds (logit) estimates. The dependent variable, contributions, is trichotomized into three categories: 0–1 coin (“low”), 2–8 coins (“some”), and 9–10 coins (“much”). Threshold coefficients (“low → some” and “some → much”) represent the estimated cutoff points between adjacent categories on the latent contribution scale. Random-effect variances indicate between-village and between-group heterogeneity (nested within villages) in the intercepts. Models 1 and 2 show results for the whole sample, whereas models 3 and 4 focus on participants who have correctly answered both control questions for understanding. The variable “FamFds” is a metric variable that includes the number of family members and friends. For the nondisclosure condition, the number of family members and friends is adjusted to be comparable to a group size of 4. P-values are shown in parentheses. Significance levels: * p < 0.10, ** p < 0.05, *** p < 0.01.*

**A.6 Analysis of variability**

***A.6.1:*** *Average contributions across villages*


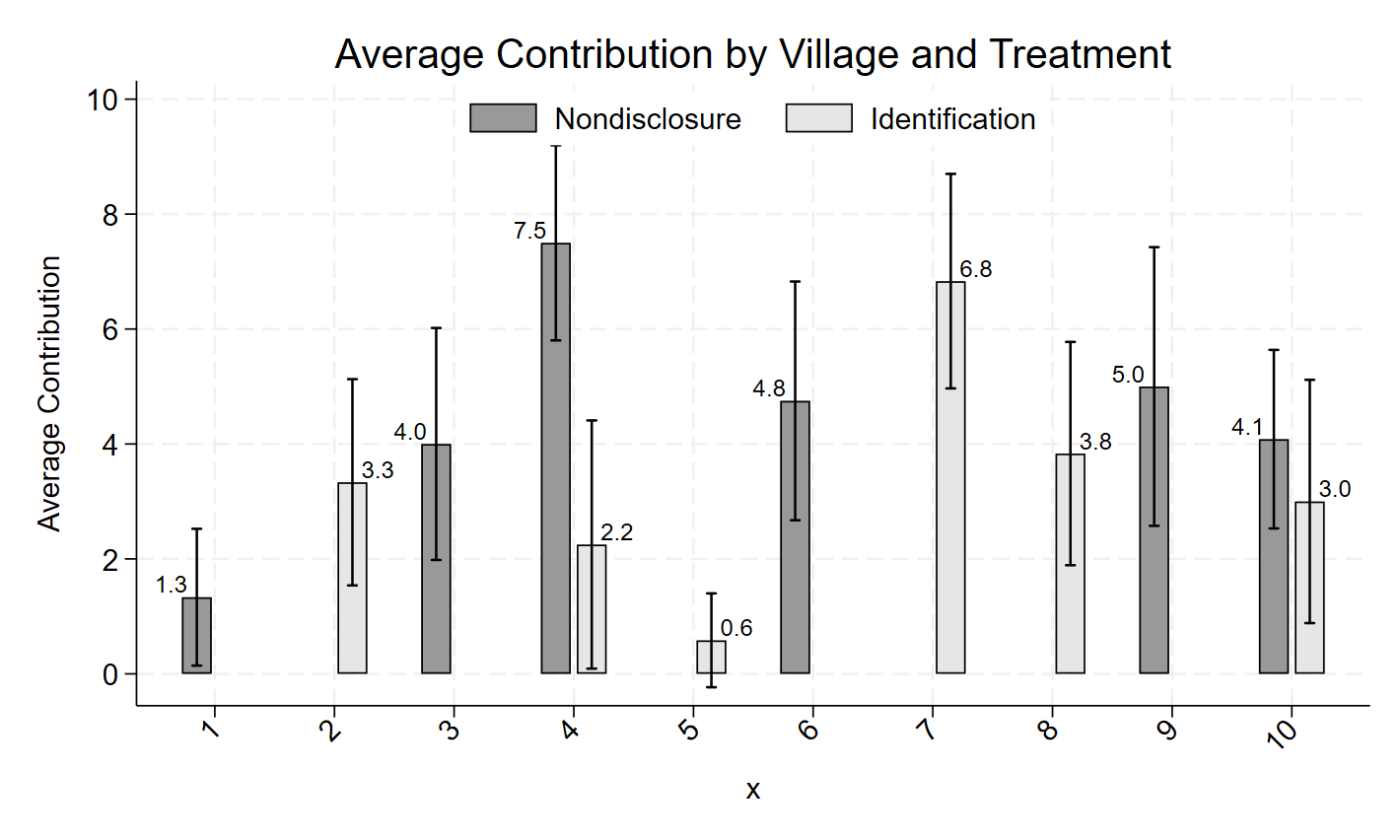


*For confidentiality, villages are not reported by name but are pseudonymized by numerical identifiers. Whiskers represent 95% Confidence intervals.*

***A.6.2****: Variation in contributions across experimental conditions*

|  | Nondisclosure | Identification | p-value for test for difference |
| --- | --- | --- | --- |
| Mean contribution | 4.444 | 3.306 |  |
| Standard deviation (overall) | 3.673 | 3.664 | 0.984 |
| Standard deviation (between groups) | 2.319 | 2.754 | 0.486 |
| Mean of standard deviation  (within groups) | 3.078 | 2.442 | 0.178 |

*sdtest for overall standard deviation and between-groups standard deviation. Two-tailed t-test for means of standard deviations within group*

**A.7 Analysis of expectations (beliefs)**

***A.7.1:*** *Distribution of expectations*


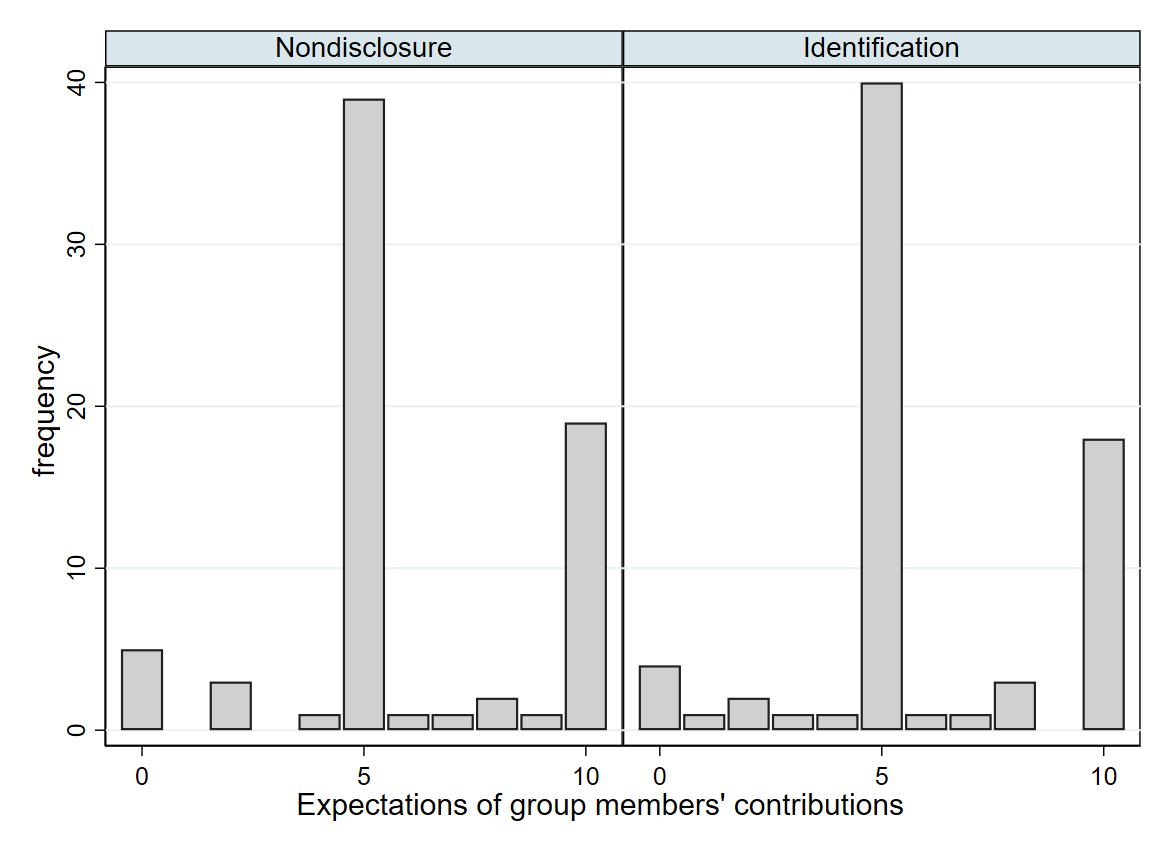


***A.7.2:*** *Correlation between expectations and contributions*

| pearson correlation coefficient  between contributions and expectations | significance (p-value) |
| --- | --- |
| 0.222 (all) | 0.008 |
| 0.254 (cqc only) | 0.006 |

*cqc = only subsample of those who correctly answered both control questions for understanding (n = 114)*

***A.7.3:*** *Multilevel ordered logit regression using expectations as the dependent variable*

| DV: expectations | **model 1** | **model 2** | **model 3** | **model 4** |
| --- | --- | --- | --- | --- |
|  | coefficient *(p)* | coefficient *(p)* | coefficient *(p)* | coefficient *(p)* |
|  |  |  |  |  |
| T: Identification | -0.132 | 0.213 | -0.228 | 0.004 |
|  | *(0.78)* | *(0.68)* | *(0.68)* | *(1.00)* |
| age |  | -0.021 |  | -0.015 |
|  |  | *(0.22)* |  | *(0.44)* |
| female (d) |  | -0.554 |  | -0.819* |
|  |  | *(0.19)* |  | *(0.10)* |
| schooling years |  | -0.083 |  | -0.078 |
|  |  | *(0.24)* |  | *(0.37)* |
| social ladder (0-10) |  | 0.070 |  | 0.102 |
|  |  | *(0.52)* |  | *(0.41)* |
| hectares (log) |  | 0.489 |  | 0.389 |
|  |  | *(0.21)* |  | *(0.42)* |
| cattle owned (log) |  | -0.121 |  | -0.106 |
|  |  | *(0.39)* |  | *(0.54)* |
| migrant (d) |  | -1.461 |  | -1.664 |
|  |  | *(0.11)* |  | *(0.13)* |
| Thresholds |  |  |  |  |
| low → some | -2.984*** | -4.136*** | -3.079*** | -4.140*** |
|  | *(0.00)* | *(0.00)* | *(0.00)* | *(0.00)* |
| some → much | 1.107*** | 0.240 | 1.148*** | 0.341 |
|  | *(0.00)* | *(0.83)* | *(0.01)* | *(0.79)* |
| Random variances |  |  |  |  |
| cons(village) | 0.210 | 0.248 | 0.185 | 0.074 |
|  | *(0.55)* | *(0.53)* | *(0.73)* | *(0.90)* |
| cons(groups) | 0.525 | 0.559 | 0.785 | 0.802 |
|  | *(0.33)* | *(0.34)* | *(0.34)* | *(0.39)* |
| N | 144 | 143 | 114 | 113 |

*Multilevel ordered logit regressions with random intercepts for villages and playing groups. Reported coefficients are log-odds (logit) estimates. The dependent variable, expectations, is trichotomized into three categories: 0–1 coin (“low”), 2–8 coins (“some”), and 9–10 coins (“much”). Threshold coefficients (“low → some” and “some → much”) represent the estimated cutoff points between adjacent categories on the latent expectations scale. Random-effect variances indicate between-village and between-group heterogeneity (nested within villages) in the intercepts. Models 1 and 2 show results for the whole sample, whereas models 3 and 4 focus on participants who have correctly answered both control questions for understanding. P-values are shown in parentheses. Significance levels: * p < 0.10, ** p < 0.05, *** p < 0.01.*

***A.7.4:*** Full models within the experimental conditions using expectations as the dependent variable (friends and family analysis)

| DV: expectations | **Nondisclosure** | **Identification** | **Nondisclosure^cqc^** | **Identification^cqc^** |
| --- | --- | --- | --- | --- |
|  | coefficient *(p)* | coefficient *(p)* | coefficient *(p)* | coefficient *(p)* |
|  |  |  |  |  |
| friends (workshop) | -0.169 |  | -0.080 |  |
|  | *(0.68)* |  | *(0.86)* |  |
| family (workshop) | -0.454 |  | -0.334 |  |
|  | *(0.20)* |  | *(0.49)* |  |
| friends (group) |  | -0.241 |  | -0.592 |
|  |  | *(0.56)* |  | *(0.70)* |
| family (group) |  | 0.467 |  | -1.290 |
|  |  | *(0.23)* |  | *(0.15)* |
| age | -0.032 | -0.051 | -0.029 | -0.163*** |
|  | *(0.18)* | *(0.21)* | *(0.28)* | *(0.01)* |
| female (d) | -1.063** | 0.447 | -0.984 | -6.392** |
|  | *(0.05)* | *(0.64)* | *(0.10)* | *(0.02)* |
| schooling years | -0.081 | -0.343* | -0.093 | -0.759*** |
|  | *(0.39)* | *(0.07)* | *(0.39)* | *(0.00)* |
| social ladder (0-10) | 0.077 | -0.179 | 0.095 | 2.232** |
|  | *(0.59)* | *(0.55)* | *(0.53)* | *(0.03)* |
| hectares (log) | 0.473 | 2.825** | 0.813 | 3.299 |
|  | *(0.32)* | *(0.02)* | *(0.14)* | *(0.23)* |
| cattle owned (log) | 0.226 | -0.836** | 0.207 | -4.531** |
|  | *(0.21)* | *(0.04)* | *(0.36)* | *(0.01)* |
| migrant (d) | -3.570 | -1.382 | -4.101 | -12.623** |
|  | *(0.16)* | *(0.40)* | *(0.12)* | *(0.02)* |
| Thresholds |  |  |  |  |
| low → some | -4.390*** | -7.511** | -3.845** | -29.503 |
|  | *(0.01)* | *(0.03)* | *(0.04)* | *(.)* |
| some → much | -0.419 | 0.382 | 0.247 | -7.242** |
|  | *(0.79)* | *(0.88)* | *(0.89)* | *(0.03)* |
| Random variances |  |  |  |  |
| cons(village) | 0.000 | 6.114 | 0.137 | 0.000 |
|  | *(1.00)* | *(0.27)* | *(0.84)* | *(1.00)* |
| cons(groups) | 0.000 | 1.765 | 0.000 | 85.112 |
|  | *(1.00)* | *(0.46)* | *(1.00)* | *(.)* |
| N | 72 | 71 | 61 | 52 |

*Multilevel ordered logit regressions with random intercepts for villages and playing groups. Reported coefficients are log-odds (logit) estimates. The dependent variable, expectations, is trichotomized into three categories: 0–1 coin (“low”), 2–8 coins (“some”), and 9–10 coins (“much”). Threshold coefficients (“low → some” and “some → much”) represent the estimated cutoff points between adjacent categories on the latent expectations scale. Random-effect variances indicate between-village and between-group heterogeneity (nested within villages) in the intercepts. Models 1 and 2 show results for the whole sample, whereas models 3 and 4 focus on participants who have correctly answered both control questions for understanding. P-values are shown in parentheses. Significance levels: * p < 0.10, ** p < 0.05, *** p < 0.01.*

***A.7.5:*** *Tables showing average expectation by number of friends and family members*

| average expectations per number of friends and family members in group | | | | |
| --- | --- | --- | --- | --- |
| number friends and family members | count nondiscl. condition | mean expectation nondiscl. | count identification condition | mean expectation identification |
| 0 | 12 | 6.42 (3.50) | 11 | 5.82 (3.43) |
| 1 | 28 | 6.11 (2.45) | 8 | 5.63 (3.20) |
| 2 | 22 | 5.91 (3.44) | 8 | 5.75 (2.82) |
| 3 | 10 | 5.50 (2.12) | 44 | 6.23 (2.50) |

*standard deviation in parentheses*

| average expectations separately per number of friends and family members in group | | | | |
| --- | --- | --- | --- | --- |
| number friends | count nondiscl. condition | mean contribution nondiscl. | count identification condition | mean identification condition |
| 0 | 34 | 6.32 (3.06) | 40 | 5.90 (2.73) |
| 1 | 31 | 5.74 (2.62) | 17 | 6.94 (2.68) |
| 2 | 6 | 5.00 (3.16) | 4 | 4.25 (1.50) |
| 3 | 1 | 10.00 (0.00) | 10 | 5.80 (2.94) |
| number family members | count nondiscl. condition | mean contribution nondiscl. | count identification condition | mean identification condition |
| 0 | 27 | 6.41 (3.10) | 24 | 5.79 (3.15) |
| 1 | 37 | 5.68 (2.78) | 15 | 5.66 (2.58) |
| 2 | 3 | 8.33 (2.89) | 10 | 6.70 (2.91) |
| 3 | 5 | 5.00 (2.12) | 22 | 6.27 (2.31) |

*There is a significant (p<0.01), negative correlation between friends and family members in one’s group. The dependence is intuitive as a higher number of one type only leaves less room for the other type in a group. Therefore, a combined measurement of friends and family members is less prone to misinterpretations due to interdependency. Standard deviation in parentheses*

**A.8 Full models within the experimental conditions** (friends and family analysis)

| DV: contributions | **Nondisclosure** | **Identification** | **Nondisclosure^cqc^** | **Identification^cqc^** |
| --- | --- | --- | --- | --- |
|  | coefficient *(p)* | coefficient *(p)* | coefficient *(p)* | coefficient *(p)* |
|  |  |  |  |  |
| friends (workshop) | 0.191 |  | -0.067 |  |
|  | *(0.64)* |  | *(0.88)* |  |
| family (workshop) | -0.124 |  | -0.131 |  |
|  | *(0.72)* |  | *(0.71)* |  |
| friends (group) |  | 0.744** |  | 0.701 |
|  |  | *(0.04)* |  | *(0.12)* |
| family (group) |  | 0.588* |  | 0.824** |
|  |  | *(0.06)* |  | *(0.04)* |
| age | 0.019 | -0.004 | 0.019 | 0.021 |
|  | *(0.40)* | *(0.86)* | *(0.42)* | *(0.46)* |
| female (d) | -0.132 | 0.918 | -0.435 | 0.487 |
|  | *(0.80)* | *(0.24)* | *(0.44)* | *(0.58)* |
| schooling years | -0.010 | -0.274** | 0.060 | -0.097 |
|  | *(0.91)* | *(0.01)* | *(0.54)* | *(0.45)* |
| social ladder (0-10) | -0.113 | -0.157 | -0.001 | 0.013 |
|  | *(0.37)* | *(0.51)* | *(0.99)* | *(0.95)* |
| hectares (log) | -0.193 | 0.332 | 0.154 | -0.578 |
|  | *(0.67)* | *(0.63)* | *(0.77)* | *(0.53)* |
| cattle owned (log) | 0.048 | 0.394 | 0.010 | 0.394 |
|  | *(0.78)* | *(0.13)* | *(0.96)* | *(0.32)* |
| migrant (d) | -0.138 | 2.202** | -0.031 | 1.811 |
|  | *(0.95)* | *(0.05)* | *(0.99)* | *(0.18)* |
| Thresholds |  |  |  |  |
| low → some | -0.865 | 0.577 | -0.043 | 2.414 |
|  | *(0.58)* | *(0.73)* | *(0.98)* | *(0.27)* |
| some → much | 1.492 | 3.424* | 2.184 | 4.883** |
|  | *(0.34)* | *(0.05)* | *(0.19)* | *(0.09)* |
| Random variances |  |  |  |  |
| cons(village) | 0.554 | 0.908 | 0.376 | 1.536 |
|  | *(0.35)* | *(0.44)* | *(0.50)* | *(0.40)* |
| cons(groups) | 0.062 | 1.366 | 0.018 | 0.000 |
|  | *(0.91)* | *(0.36)* | *(0.97)* | *(1.00)* |
| N | 72 | 71 | 61 | 52 |

*Multilevel ordered logit regressions with random intercepts for villages and playing groups. Reported coefficients are log-odds (logit) estimates. The dependent variable, contributions, is trichotomized into three categories: 0–1 coin (“low”), 2–8 coins (“some”), and 9–10 coins (“much”). Threshold coefficients (“low → some” and “some → much”) represent the estimated cutoff points between adjacent categories on the latent contribution scale. Random-effect variances indicate between-village and between-group heterogeneity (nested within villages) in the intercepts. Models 1 and 2 show results for the whole sample, whereas models 3 and 4 focus on participants who have correctly answered both control questions for understanding. P-values are shown in parentheses. Significance levels: * p < 0.10, ** p < 0.05, *** p < 0.01.*

***A.9:*** ***Average contributions by number of friends and family members***

| average contributions per number of friends and family members in group | | | | |
| --- | --- | --- | --- | --- |
| number friends and family members | count nondiscl. condition | mean contribution nondiscl. | count identification condition | mean contribution identification |
| 0 | 12 | 4.83 (4.11) | 11 | 1.45 (3.21) |
| 1 | 28 | 3.96 (3.46) | 8 | 3.13 (3.56) |
| 2 | 22 | 4.68 (4.16) | 8 | 1.88 (3.36) |
| 3 | 10 | 4.80 (2.90) | 44 | 4.14 (3.70) |

*Standard deviation in parentheses*

| average contributions separately per number of friends and family members in group | | | | |
| --- | --- | --- | --- | --- |
| number friends | count nondiscl. condition | mean contribution nondiscl. | count identification condition | mean contribution identification |
| 0 | 34 | 3.97 (3.70) | 40 | 3.33 (3.54) |
| 1 | 31 | 5.06 (3.72) | 17 | 3.18 (3.99) |
| 2 | 6 | 3.00 (2.28) | 4 | 4.50 (4.04) |
| 3 | 1 | 10.00 (0.00) | 10 | 3.30 (4.00) |
| number family members | count nondiscl. condition | mean contribution nondiscl. | count identification condition | mean contribution identification |
| 0 | 27 | 5.19 (3.86) | 24 | 2.33 (3.47) |
| 1 | 37 | 4.00 (3.51) | 15 | 3.20 (3.93) |
| 2 | 3 | 3.33 (5.77) | 10 | 3.80 (4.16) |
| 3 | 5 | 4.40 (2.88) | 22 | 4.36 (3.40) |

*There is a significant (p<0.01), negative correlation between friends and family members in one’s group. The dependence is intuitive as a higher number of one type only leaves room for fewer of the other type in a group. Therefore, a combined measurement of friends and family members is less prone to misinterpretations due to interdependency.*

*Standard deviation in parentheses*

**Supplement B: Experimental Protocols**

*B.1 Village meeting*

| **[VILLAGE MEETING]** ***[freely presented by Christian, interpreted by Moses (assistant)]***  To begin with, we would like to thank you all for coming here today. My name is Christian Hoenow. I am from the University of Marburg in Germany. Together with the Ministry of Agriculture, Water and Forestry we are conducting research under the SASSCAL research project. [NAME OF EXPRIMENTERS] are also part of the project.  Doing research means we are just here to collect data, but we do not bring any type of development project into the village. What you answer in the workshop will not have any impact on future projects.  Today we would like to conduct two small workshops with a certain number of people. During this workshop we will also ask you several questions. Unfortunately, not everyone from this village can participate since each workshop can only include a certain number of participants.  Since we want everyone to have the same chance to participate, we have prepared a bag with as many cards as people present. Each adult that is older than 18 years now will draw a card. We will ask you to fully concentrate on the workshop and we will be asking many questions. If you already know that you cannot attend for up to 5 hours, or do not wish to answer many questions, you should please not draw. Participation is, of course, voluntary!   - If you draw a red card, you will participate in the first workshop, which is conducted by Christian - If you draw a blue card, you will participate in the second workshop, which is conducted by Adrian - If you draw a white card, you unfortunately cannot participate in any of the events.   Do you have any questions?  **[let every adult draw a card]**  Now that everyone has drawn a card, we would like to ask all participants with a white card to leave the area. Thank you very much for attending the meeting.  Those who have drawn a blue card, please follow Adrian. He will right away start with the meeting.  **[wait for everyone to leave except Christian’s participants]**  **[continue with general instructions in each condition]** |
| --- |

*B.2 General instructions*

| **[GENERAL INSTRUCTIONS]** To begin with, we would like to thank you all for coming here today.  We will conduct a workshop and at the end you will receive some payment for participating. The money is not our private money, but it is provided by the German government.  All information collected today will be used for research only. Neither the government of Namibia, Germany nor any other organization will receive the data for other purposes. Also, neither your names nor any village-specific information will be linked to the results. All answers will remain anonymous to others.  The schedule for today looks as follows:   1. We will explain the procedure of the workshop. 2. We will conduct a small workshop. 3. After the games each of you answers a short questionnaire. 4. Finally, you will receive the money.   Before starting, I would like to give you some general information:   1. If at any time, you think that this is something that you do not wish to participate in for any reason, you are free to leave. You will however only receive a payment if you stay until the end of the workshop. 2. If you already know that you will not be able to stay for at least 5 hours, then you should leave right away. 3. We require your complete and undistracted attention. Please, follow the instructions carefully and do not use your phone or engage in any other distracting activity. 4. It is not allowed to talk to each other during the workshop unless we tell you to. You can ask questions after raising your hand. If you talk to each other when you are not allowed to, you will be excluded from the workshop and the payments. 5. Every one of you has received a unique ID card. Please keep this ID until the end. You must return the ID before receiving the money at the end of the workshop.   After knowing these rules, is there anybody who does not want to participate?  Do you have any questions?  **[continue with experimental conditions]** |
| --- |

*B.3 Game instructions public good game*

| **[GAME INSTRUCTIONS]**  We will now explain the procedure of the workshop. Please pay attention as for participating it is necessary that you understand everything. Also, we will later ask you questions individually to check whether you understood everything correctly. Each one of you will now receive an envelope that contains 10 Experimental Coins (EC). Each EC is worth 5 N$. **[show coins]**. You will have to decide whether to contribute that money to a group account or not. What you put in the brown envelope is what you want to contribute to the group account, whereas what you wish to keep must be put in the white envelope **[show envelopes]**.  You can contribute any amount between 0 and 10 EC. The coins that you do not contribute are yours and you can keep them for sure. After the game we will change them for you: 5N$ for every EC. [See graph with exchange rate] In total you can get between 25 and 125N$, depending on your decisions and the decision of the other players in your group. You are playing the game with three other players, i.e., in groups of four.   - The number of ECs that were contributed to the group account are doubled. This doubled amount is then equally divided by all four players in the group. - That means every player receives on fourth of the doubled group account. - In total you will earn the ECs that you keep plus the share that you receive from the group account - Note that the game is not about luck and not about being better than others. Everyone will receive exactly the amount as determined by the rules explained. |
| --- |

*B.4 Poster*

| 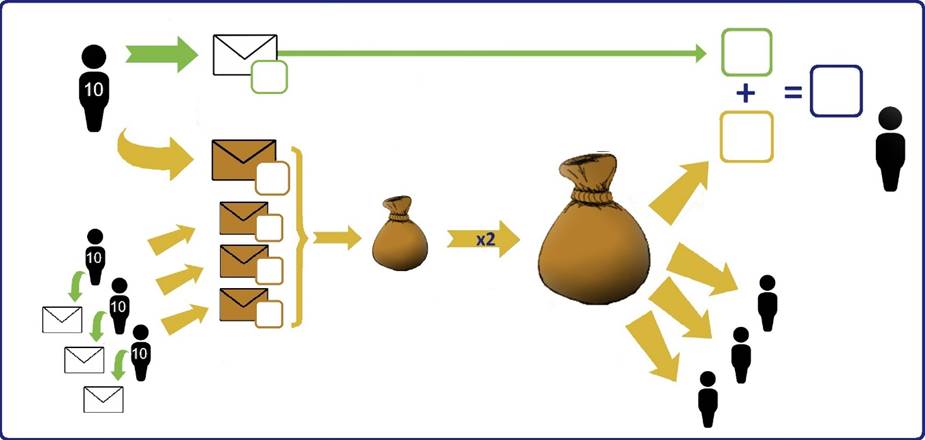 |
| --- |

*B.5 Examples*

| Example 1  **[use poster and fill with example numbers]**  4 players contribute half of their endowment to the group account.   - There are then 5 x 4 = 20EC in the group account. - The 20EC in the group account are then doubled (20EC x 2 = 40 EC) and divided equally to all 4 players. - This means each player in the group receives 40EC / 4 = 10 EC from the group account.   Each player then ends up with the amount that he/she kept, which is 5EC and the amount that he/she received from the group account, which is 10EC. In total it results in 15EC for all players. |
| --- |

| Example 2  **[use poster and fill with example numbers]**  3 players contribute all of their ECs to the group account and 1 player does not contribute anything.   - There are then 30EC in the group account. - The 30EC in the group account are then doubled (30EC x 2 = 60 EC) and divided equally to all 4 players. - This means each player in the group receives 60EC / 4 = 15 EC from the group account.   The one player that did not contribute receives 15EC from the group account plus 10EC that he/she kept for himself, which is 25 EC in total.  The three players that contributed everything receive 15 EC each from the group account. |
| --- |

| Example 3  **[use poster and fill with example numbers]**  Imagine now that the one player also contributes. So, everyone contributes everything.   - Then the total contributions are 4x10 = 40. Multiplied by 2 = 80. 80 divided equally amongst all four players is 20EC for everyone. - Then the three players get 20 instead of 15, and the one player who now also contributed also receives 20, instead of the 25 he/she would receive if NOT contributing. |
| --- |

*B.6 Control questions for public use*

| We would now like to ask you a few questions to check if everybody understood:  **[try to involve all participants]**   1. If no one contributes anything, that means everyone keeps his/her initial ECs. Then how much does every player end up with?   **[10]**   1. If everyone contributes all of his/her initial ECs, then how much does every player get? **[20]** 2. Are the payoffs for everyone higher, lower or the same if all 4 players contribute 8EC, compared to when all players contribute 5EC?   **[higher]**   1. If you do not contribute anything are your own payoffs higher, lower or the same compared to when you contribute?   **[always higher]**   1. What is your payoff if you contribute all of your 10EC but no one else contributes anything?   **[then only 10EC in the group account, 10*2 = 20, divided by four = 5ECs for everyone. Since you did not keep any of your initial EC, your final payoff is 5EC.]**   1. How much does everyone else receive in this case?   **[keep 10 for themselves + get 5 from your contribution = 15]**   1. If you end up with 10 coins, how many N$ will you get for that later?   **[5 x 10 = 50 N$]**   - Very good. Is there anything unclear about the rules or how the payoffs are calculated? - Should we have another example? |
| --- |

*B.7 Experimental conditions*

| **[CONDITION 1: NONDISCLOSURE]**  **[read Game Instructions]**  In the following game you will be playing in groups of 4 players. As you see, we are 12 players here. Therefore, we will have 3 groups playing the game simultaneously. But you do not know who your three team members are. It will not be revealed after the game is over, either. The other groups are playing the same game, but what they do does not influence your group or your payoffs. They do just play the same game simultaneously. The group allocation is entirely random according to your ID numbers.  Remember that you are not allowed to talk to each other.  **[then wait for a short moment in silence before starting the game]** |
| --- |

| **[CONDITION 2: IDENTIFICATION]**  **[read Game Instructions]**  In the following game, we will divide you into groups of four. That means each of you is playing the game with three other players. The other groups are playing the same game, but what they do does not influence your group or your payoffs. They do just play the same game simultaneously.  **[allocate groups]**  **[groups should sit together, but keep distance to avoid communication between groups]**  This is the group you will be playing with. Remember that you are not allowed to talk to each other.  **[then wait for a short moment in silence before starting the game]** |
| --- |

*B.8 Decision making*

| **[DECISION MAKING]**  Your contribution will not be disclosed to the other participants. You will find out about the total contributions in your group at the end when we pay you, but no one will find out about how much other single players contributed. That means your own contribution is also anonymous to the other players. We will not disclose your decisions and you are under no obligation to tell anyone about how much you contributed.  In order to ensure anonymity in decision making, you will one-by-one come to the booth and make the decision there, in private. Please do not show other players how much you contributed, also not after you have made your contribution decision.  **[show both envelopes and how to do it]**   - Are there any questions about the procedure?   Before we start with the decisions, we would like to ask you two control questions, in order to check whether you have really understood the game. The answers you give here will not affect the money you earn; it is just for us as additional feedback information. [Assistant (me)] will ask you these questions, then you go directly to the booth and make your decision, then put the brown envelope, which contains your contribution to the group account into the box.  Please now come to the booth. We will call you one by one. Please remember to not talk to each other or communicate in any other way while waiting until everyone has made their decision. Also remember that there is no right or wrong in this game.  After the decision you may directly move to the snack area. There, you may talk again freely.  **[one-by-one to assistant to answer two control questions in private, then to booth to make decision, in convenient order]**  **[have a break with snacks and cold drinks for everyone]**  Thank you all for participating. You will now answer some short questionnaire and afterwards you will get the payments. |
| --- |

*B.9 Private Control Questions and Expectations*

| **[CONTROL QUESTIONS FOR UNDERSTANDING**  **AND COLLECTION OF EXPECTATION]**  We would now like to ask you two questions for understanding of the game mechanics in private. Whether you answer these two questions correctly or not will not affect your payoff. You will still be participating in the game, no matter your answer. It is just for us to learn if our explanations were understandable.  **[Question 1]**   - If the **other three players each contribute 5 ECs** to the group account and **you contribute 10 ECs**, then **who earns more** in the end? You, each other player or all the same?   [correct answer: each other player]  **[Question 2]**   - If the **other three players each contribute 10 ECs** to the group account and **you contribute 5 ECs**, then **who earns more** in the end? You, each other player or all the same?   [correct answer: you]  Next, we would like to learn about **your expectation** of your three group members’ average contribution. How many out of their 10 ECs do you believe **your three group members** will contribute to the group account, **on average**? You will be **awarded an additional 20N$** if your guess turns out as correct later on. Your guess will be counted as correct if it is within a range of 1N$ of the actual average. **[Please make a decision within 1/2 minute]**  **[Expectation / Belief]**   - Your expectation: __________ ECs [0-10]   Thank you very much! You may now proceed to make your decision at **[point toward secluded location for decision making].** |
| --- |

**Supplement C: Survey Questions** (Sheets produced with Kobo-Toolbox: “https://www.kobotoolbox.org/”)


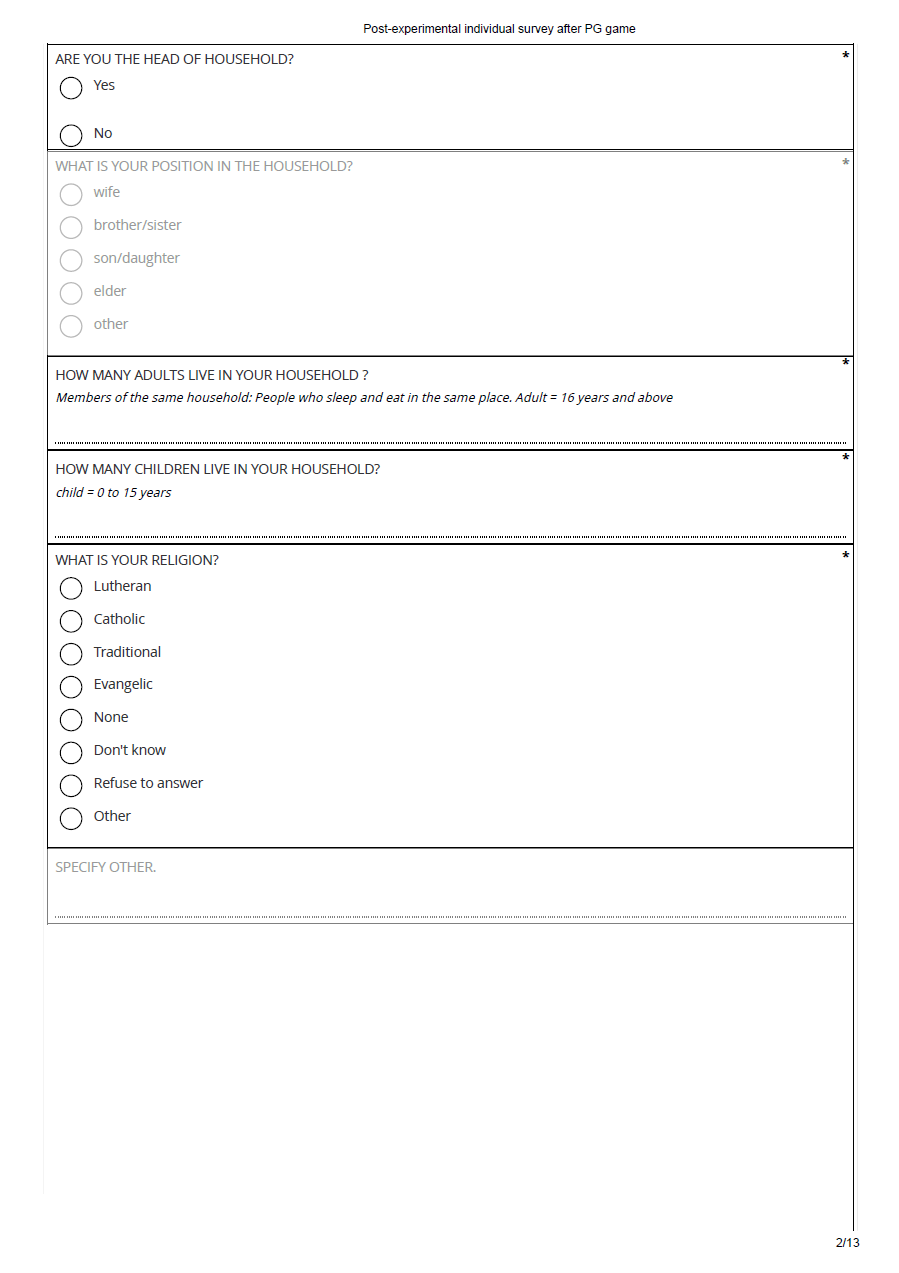


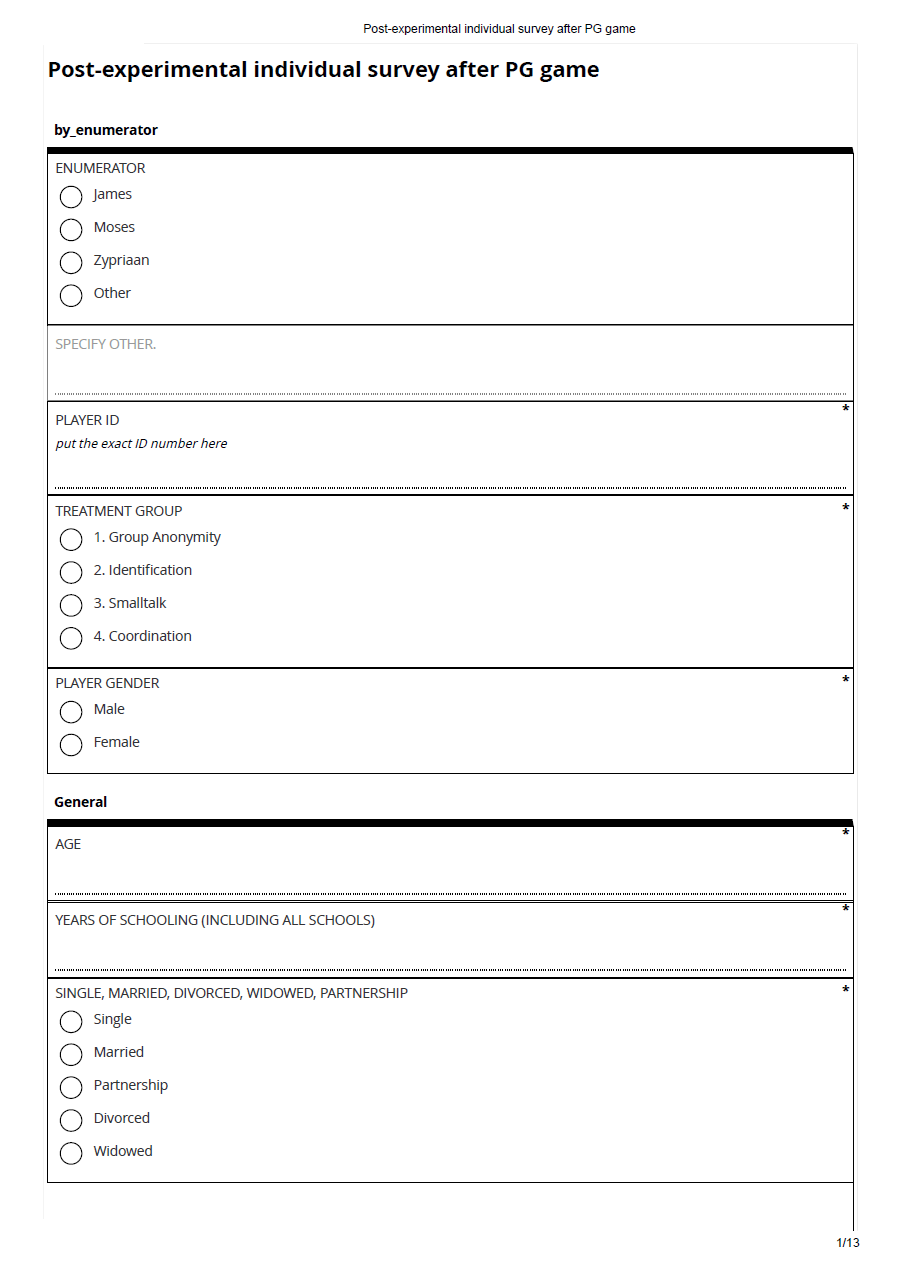


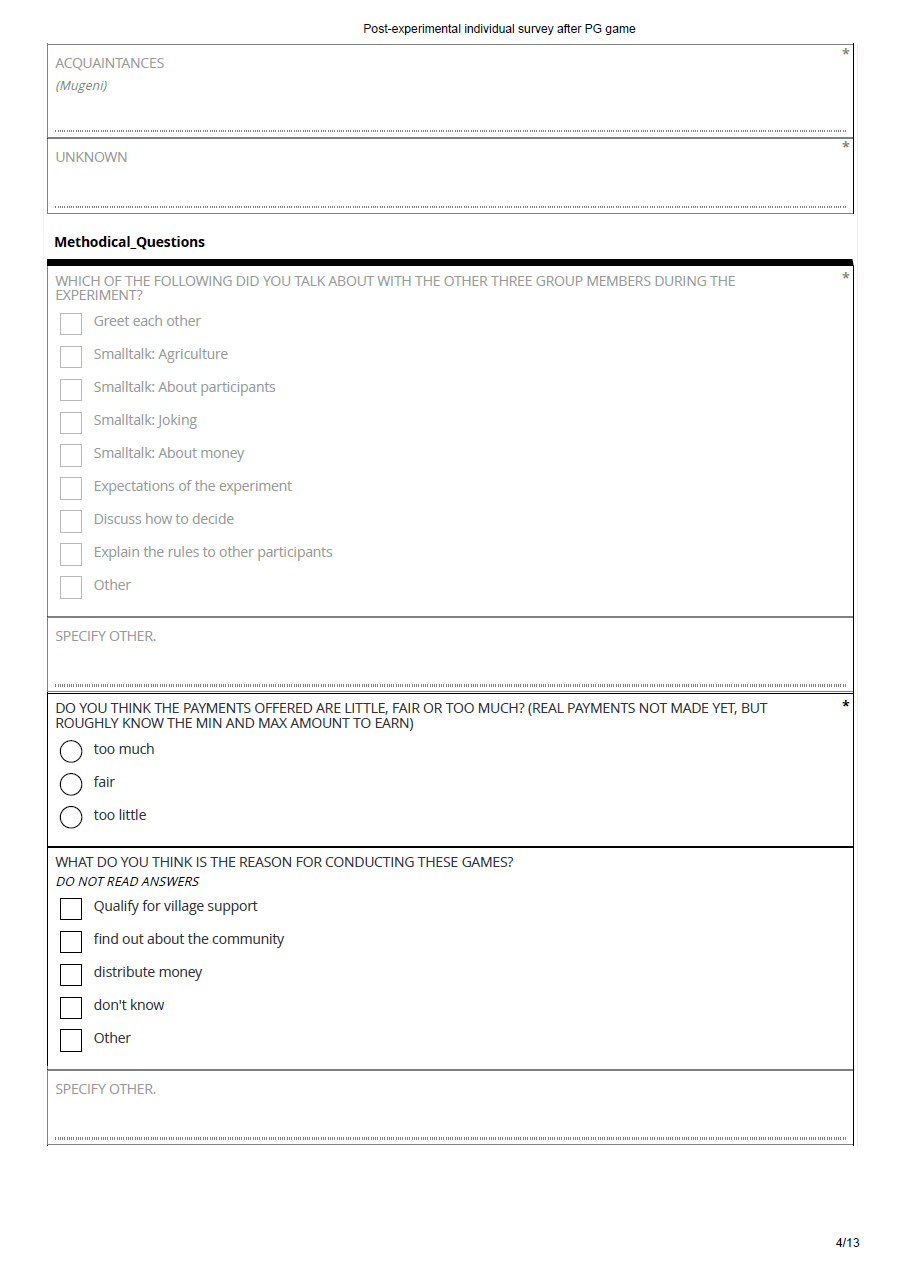


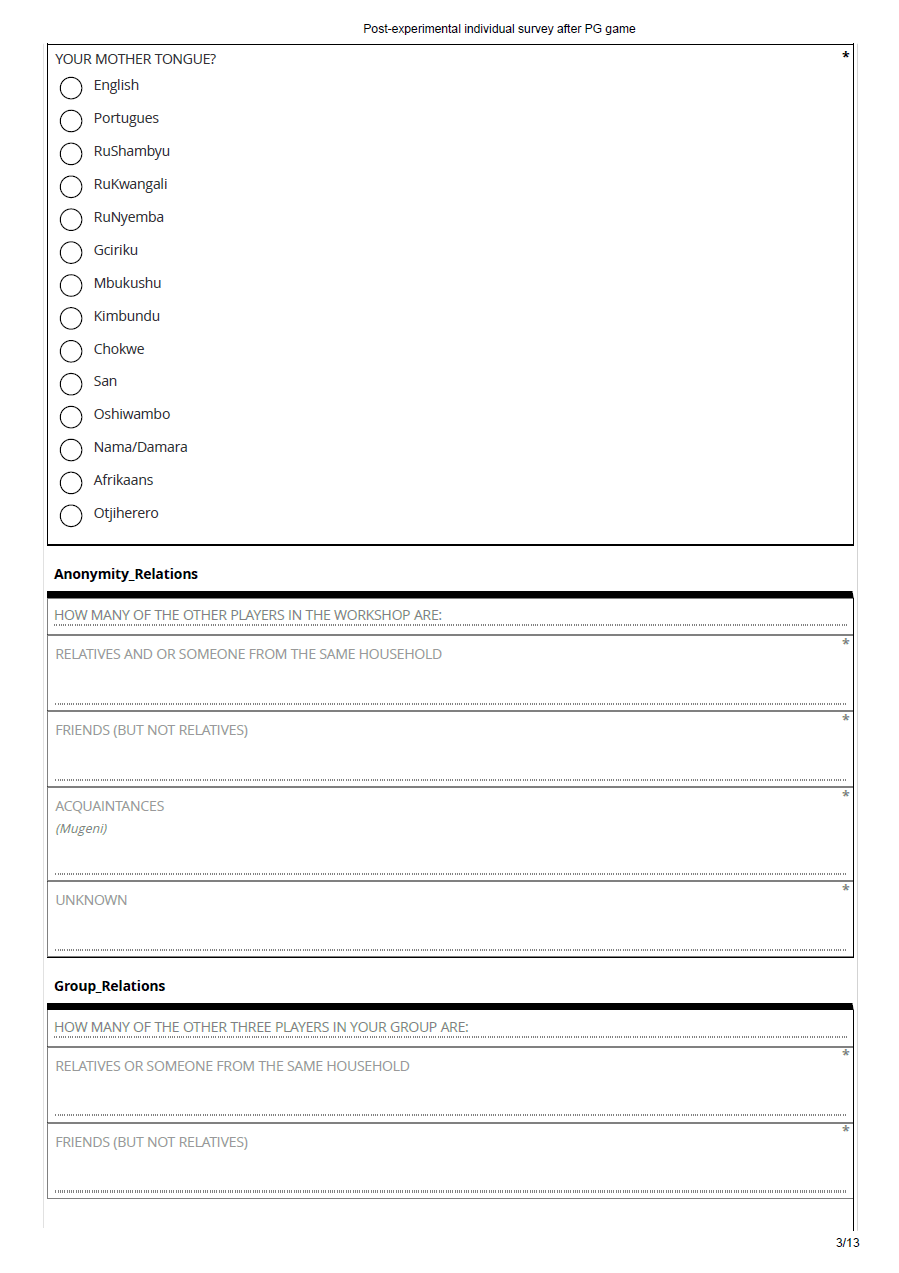


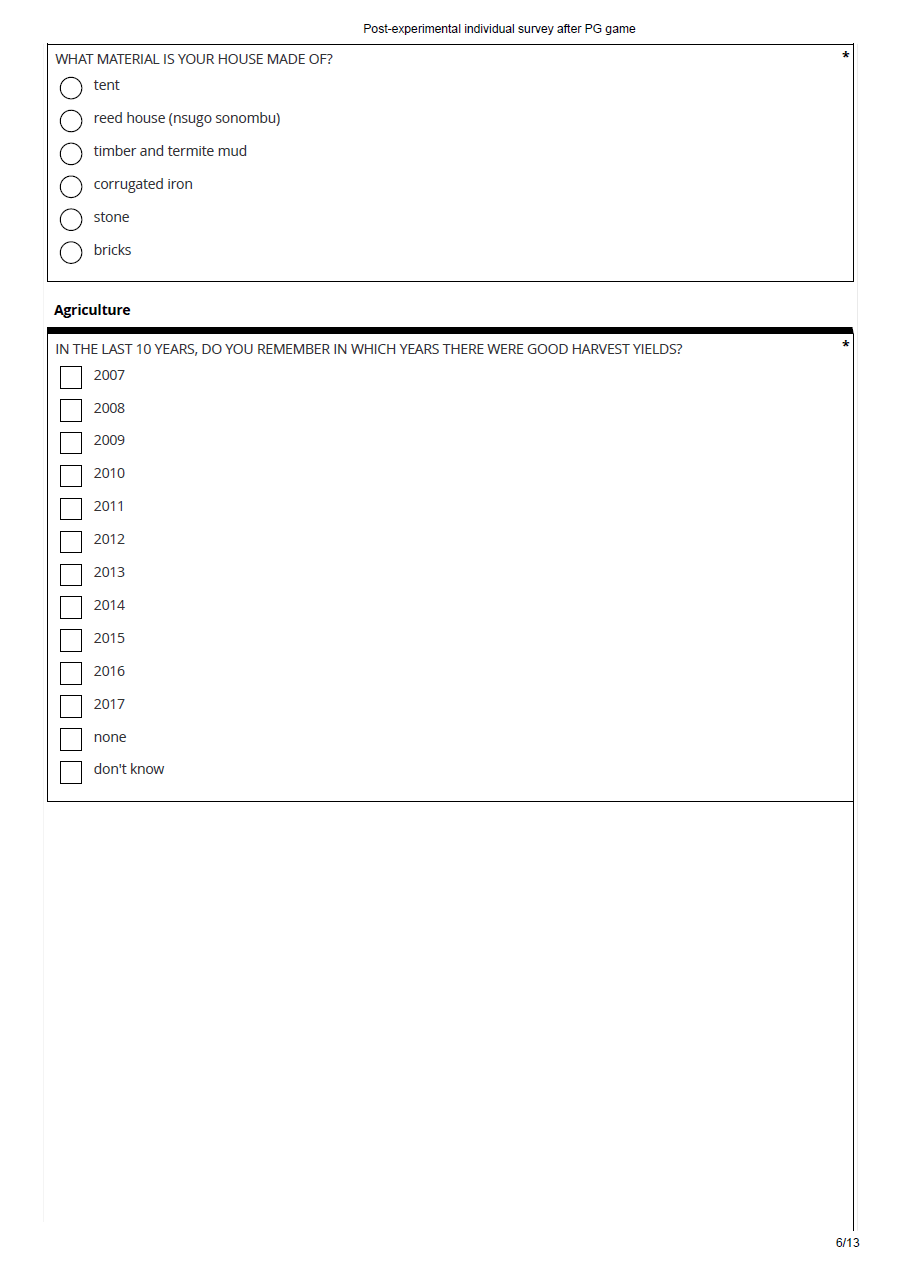


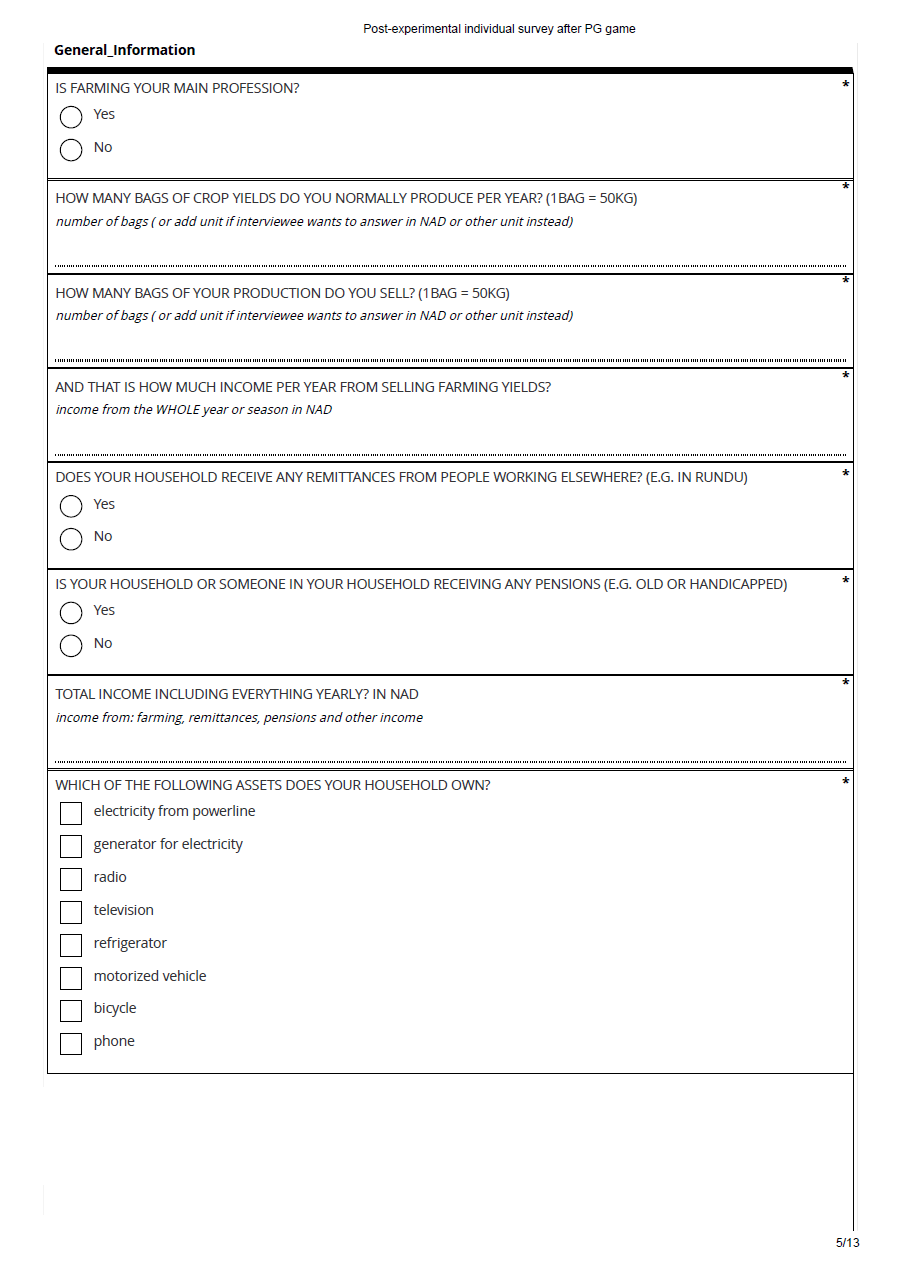


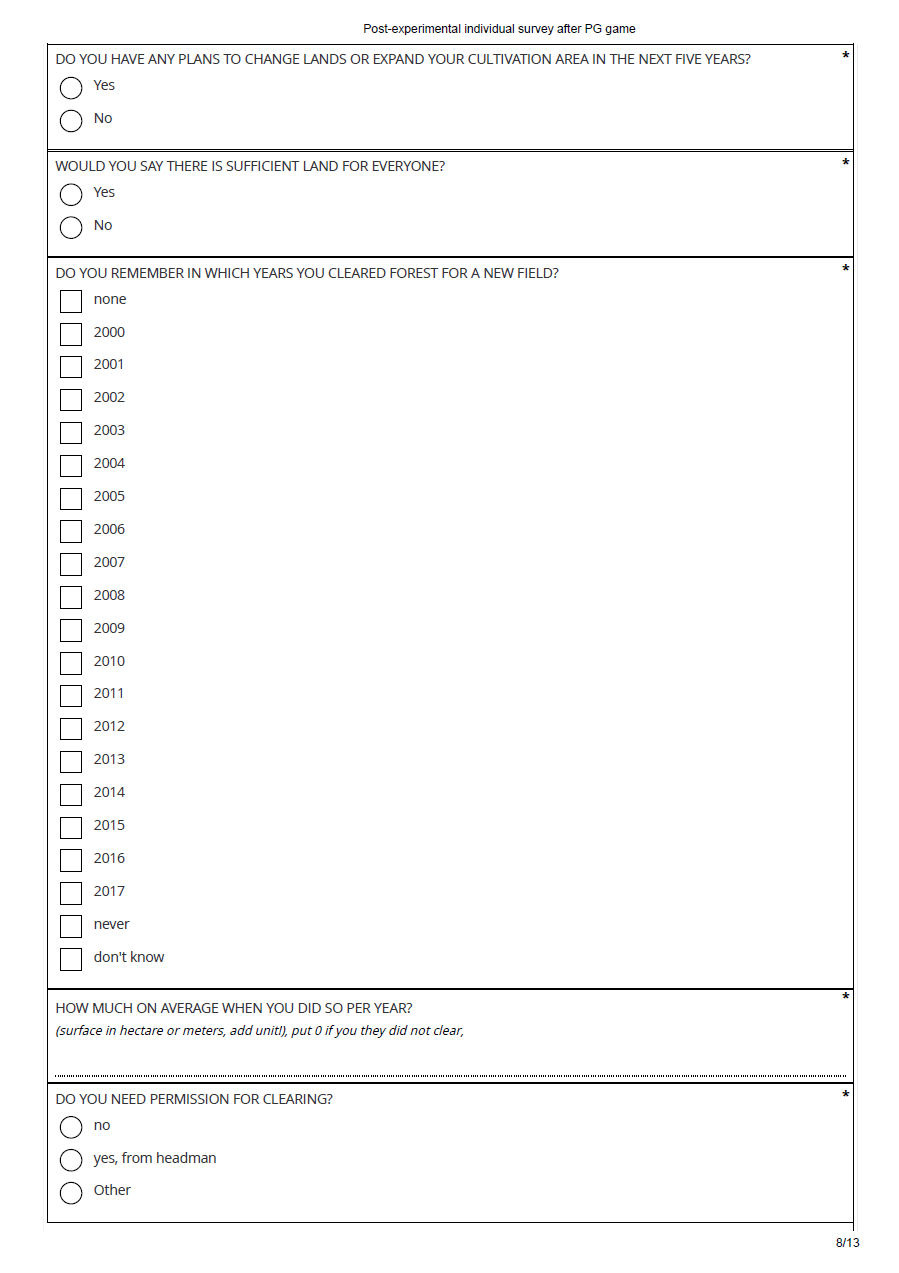


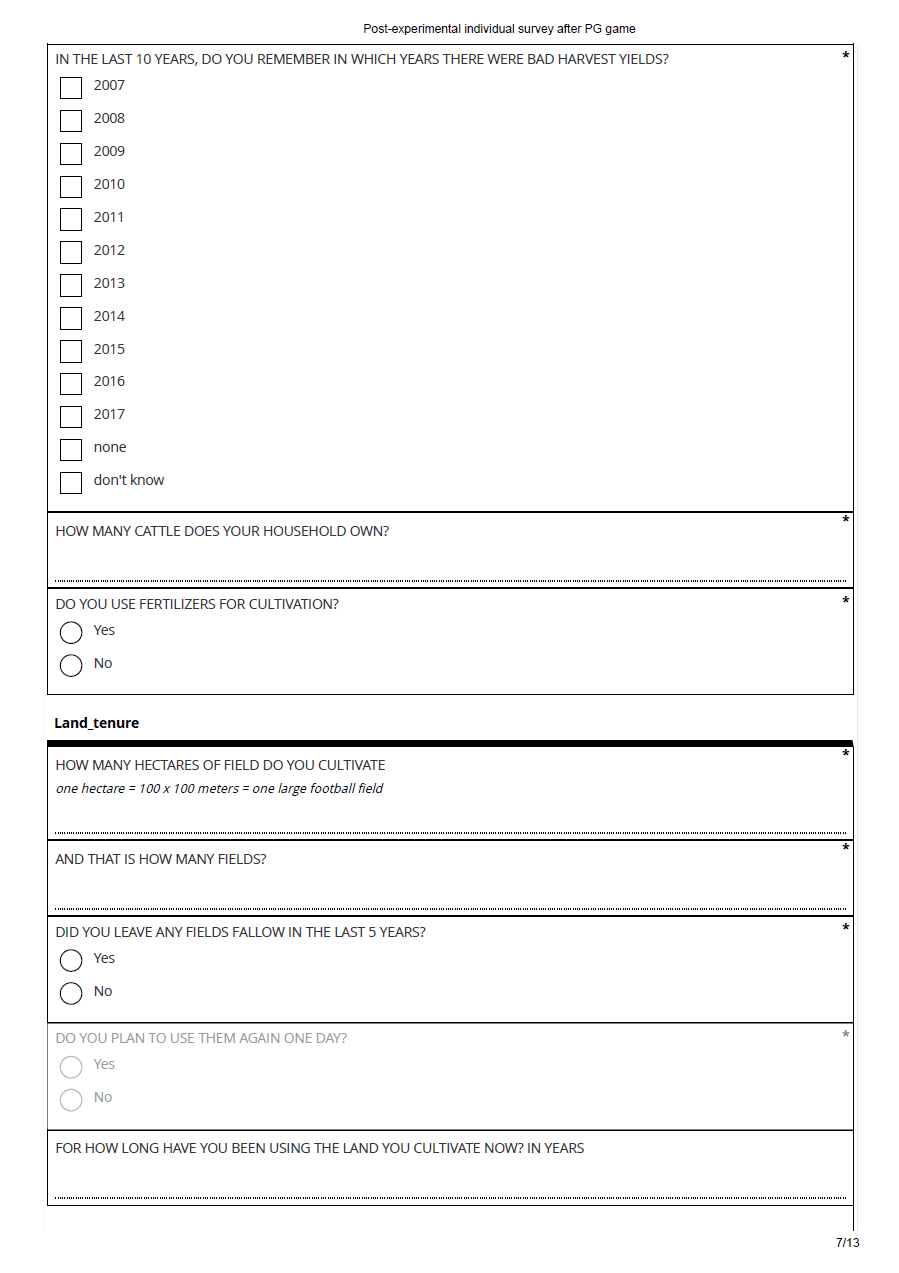


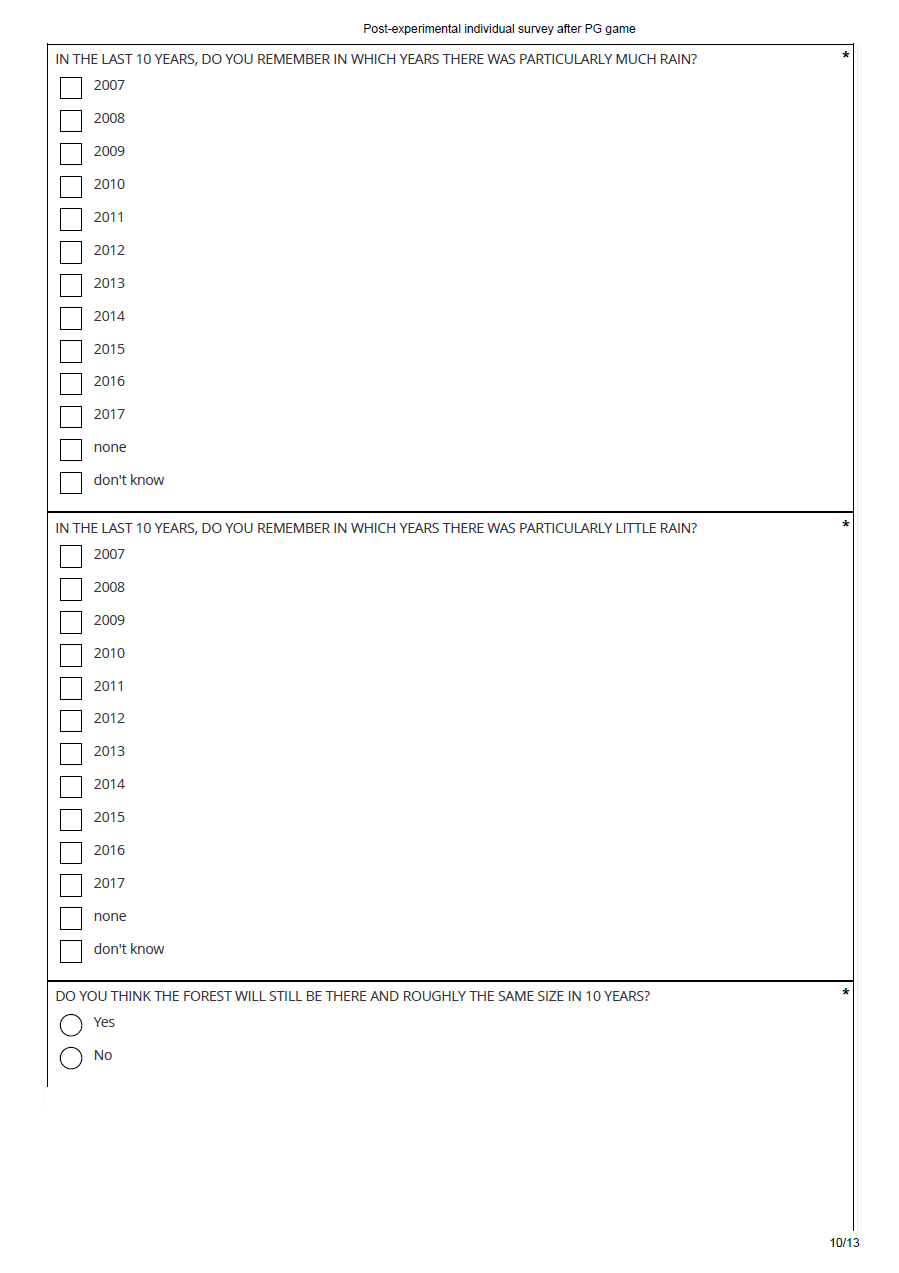


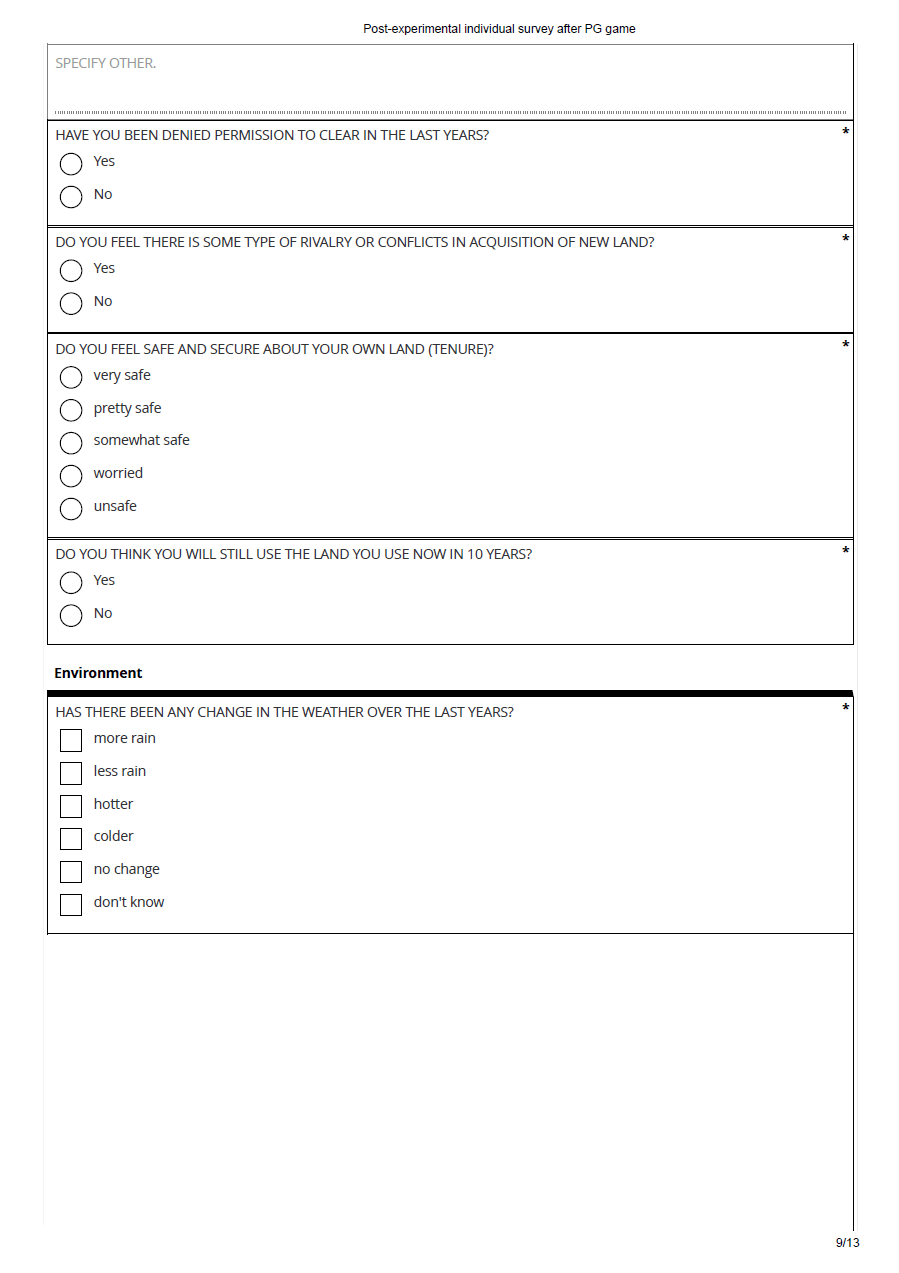


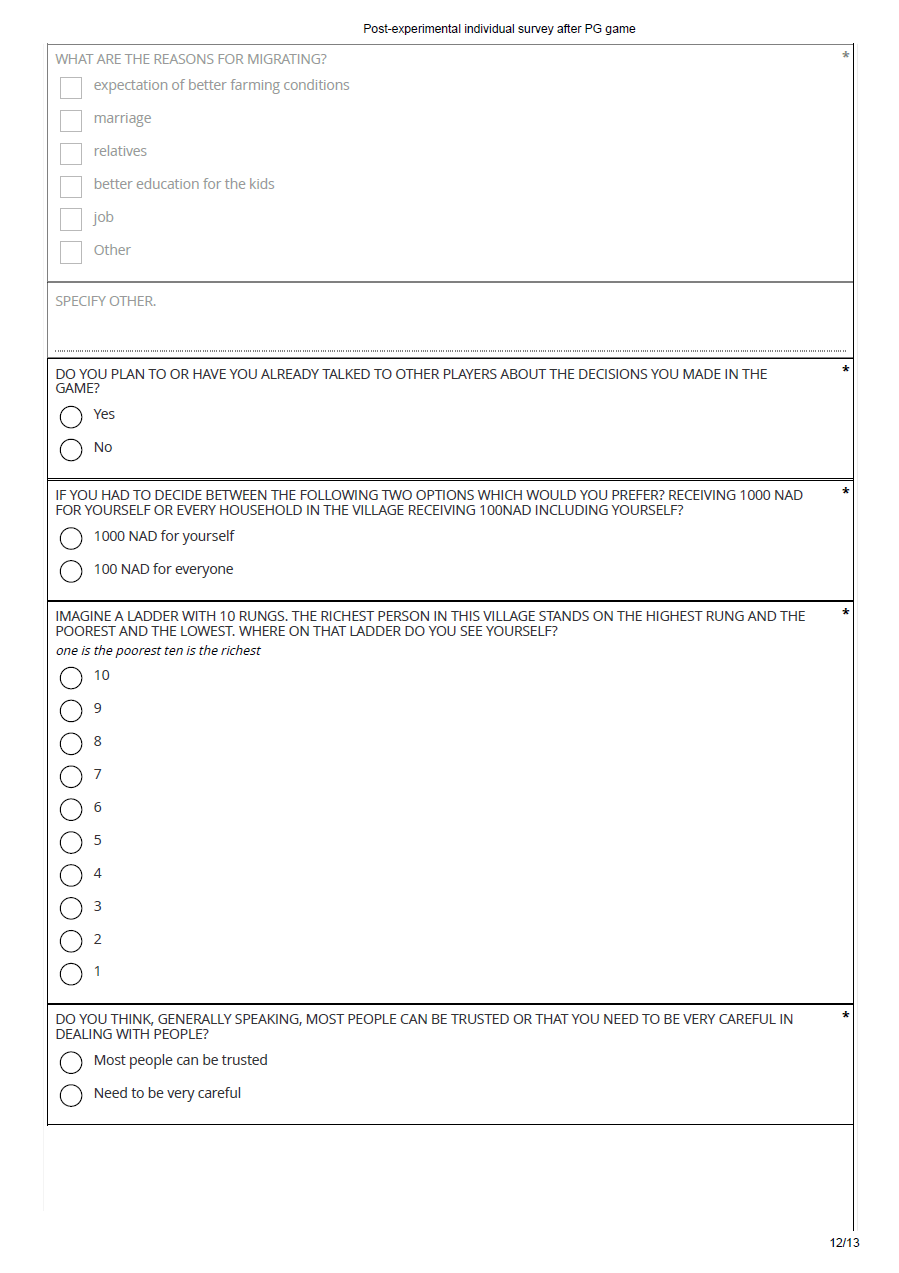


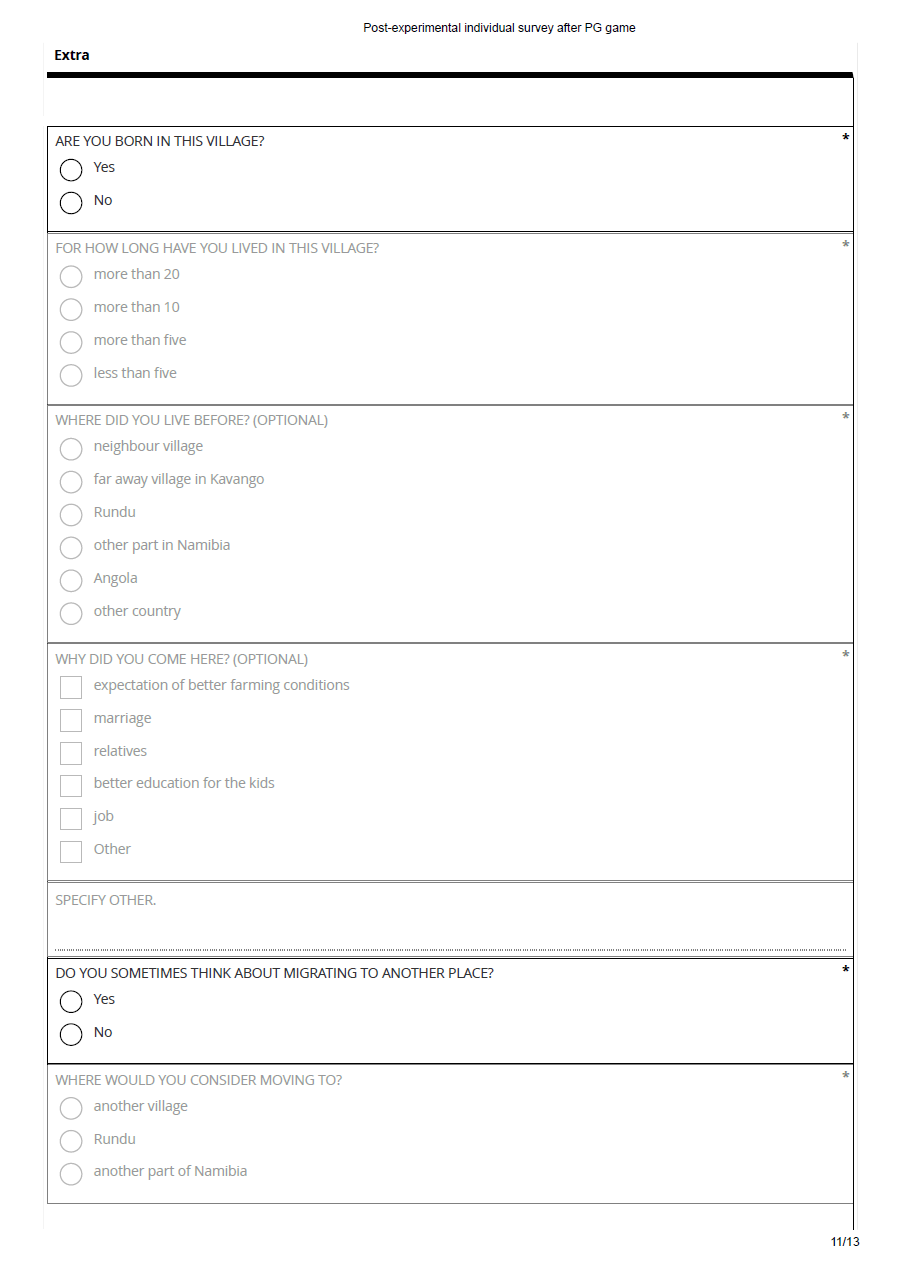


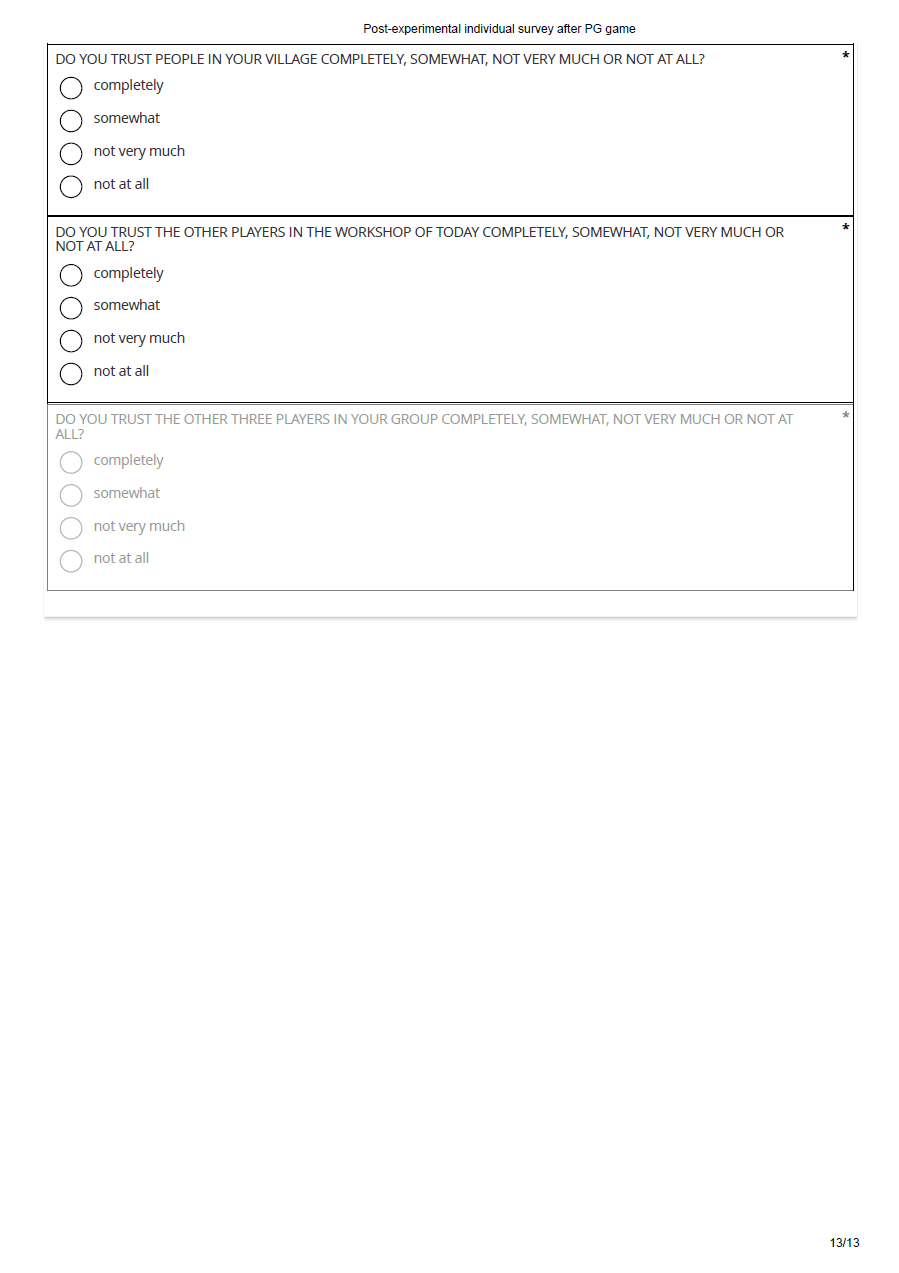

Supplement: Supplementary file 1 — Supplementary file1 (DOCX 1053 KB) [file 12110_2025_9508_MOESM1_ESM.docx]
